# Synthesis and reactivity of *cyclo*-tetra(stibinophosphonium) tetracations: redox and coordination chemistry of phosphine–antimony complexes†
†Electronic supplementary information (ESI) available. See DOI: 10.1039/c4sc03939d



**DOI:** 10.1039/c4sc03939d

**Published:** 2015-02-03

**Authors:** Saurabh S. Chitnis, Alasdair P. M. Robertson, Neil Burford, Jan J. Weigand, Roland Fischer

**Affiliations:** a Department of Chemistry , University of Victoria , Victoria , BC V8W 3V6 , Canada . Email: nburford@uvic.ca ; Fax: +1 250 721 7147 ; Tel: +1 250 721 7150; b Department of Chemistry and Food Chemistry , TU Dresden , 01062 , Dresden , Germany . Email: jan.weigand@tu-dresden.de ; Tel: +49 351 46842800; c Institute for Inorganic Chemistry , TU Graz , 98010 , Graz , Austria . Email: roland.fischer@tugraz.at ; Tel: +43 316 87332109

## Abstract

Reductive elimination of [R_3_PPR_3_]^2+^, [**11(R)**]^2+^, from the highly electrophilic Sb^III^ centres in [(R_3_P)_3_Sb]^3+^, [**8(R)**]^3+^, gives Sb^I^ containing cations [(R_3_P)Sb]^1+^, [**9(R)**]^1+^, which assemble into frameworks identified as *cyclo*-tetra(stibinophosphonium) tetracations, [(R_3_P)_4_Sb_4_]^4+^, [**10(R)**]^4+^. A phosphine catalyzed mechanism is proposed for conversion of fluoroantimony complexes [(R_3_P)_2_SbF]^2+^, [**7(R)**]^2+^, to [**10(R)**]^4+^, and the characterization of key intermediates is presented. The results constitute evidence of a novel ligand activation pathway for phosphines in the coordination sphere of hard, electron deficient acceptors. Characterization of the associated reactants and products supports earlier, albeit less definitive, detection of analogous phosphine ligand activation in Cu^III^ and Tl^III^ complexes, demonstrating that these prototypical ligands can behave simultaneously as reducing agents and σ donors towards a variety of hard acceptors. The reactivity of the parent *cyclo*-tetra(stibinophosphonium) tetracation, [**10(Me)**]^4+^, is directed by high charge concentration and strong polarization of the P–Sb bonds. The former explains the observed facility for reductive elimination to yield elemental antimony and the latter enabled activation of P–Cl and P–H bonds to give phosphinophosphonium cations, [Me_3_PPR′2]^1+^, including the first example of an H-phosphinophosphonium, [(Me_3_P)P(H)R′]^1+^, and 2-phosphino-1,3-diphosphonium cations, [(Me_3_P)_2_PR′]^2+^. Exchange of a phosphine ligand in [**10(Me)**]^4+^ with [*nacnac*]^1–^ gives [(Me_3_P)_3_Sb_4_(*nacnac*)]^3+^, [**15(Me)**]^3+^, and with *dmap* gives [(Me_3_P)_3_Sb_4_(*dmap*)]^4+^, [**16**]^4+^. The lability of P–Sb or Sb–Sb interactions in [**10(Me)**]^4+^ has also been illustrated by characterization of heteroleptically substituted derivatives featuring PMe_3_ and PEt_3_ ligands.

## Abstract 2

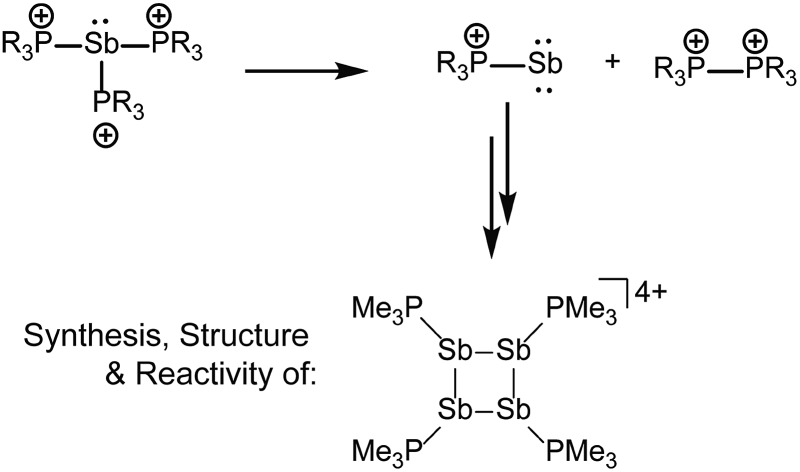
Reactions of trialkylphosphines with antimony(iii) triflates yield *catena*-antimony(i) cations revealing a new reductive elimination/oxidative coupling reaction for P–Sb coordination complexes.

## Introduction

**Scheme 1:**
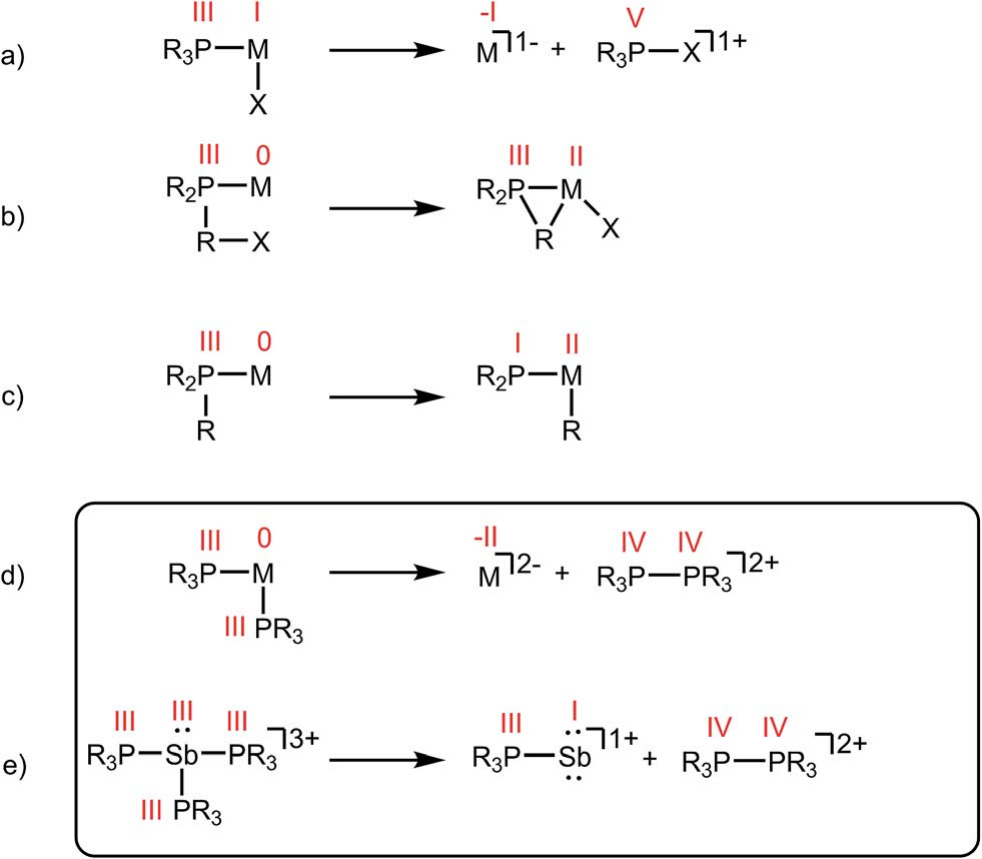
Activation of phosphine ligands in the coordination sphere of a Lewis acceptor. Numerals in red denote formal oxidation states for the element.

Phosphines are prototypical ligands in the coordination chemistry of d-block metals. While the chemistry of p-block elements is primarily defined by covalent bonding as typified by organic frameworks, an array of phosphine adducts has also been characterized for main group element acceptors.^1–5^ Beyond their versatile ligand properties as neutral, two-electron donors (L-type),^6^ phosphines also exhibit redox reactivity within the coordination sphere of an acceptor. For example, reductive elimination of tetraorgano- or halotriorganophosphonium cations (Scheme 1a),^7^ and oxidative addition of PR–X bonds (Scheme 1b),^8^ or P–R bonds (Scheme 1c)^9^ are all known pathways of tertiary phosphine activation in transition metal chemistry. One report^10^ hints at the reductive elimination of a diphosphonium dication from a phosphine–metal complex (Scheme 1d). In this instance, spectroscopic studies indicate that the reaction of excess PMe_3_ with [Cu(MeCN)_*x*_][PF_6_]_2_ or [Tl(MeCN)_*x*_][UF_6_]_3_ yields [Me_3_PPMe_3_]^2+^, and the reduced metal complexes [Cu(PMe_3_)_4_][PF_6_] and [Tl(PMe_3_)_2_][UF_6_], respectively.^10^ However, neither the high oxidation state reactants nor the reduced products have been structurally verified and three different ^31^P NMR chemical shifts were ascribed to [Me_3_PPMe_3_]^2+^ (depending upon the counterion: +65.0 ppm, +46.3 ppm, or +27.8 ppm). As reductive elimination is observed for both a transition metal (Cu^II^) and a main group metal (Tl^III^) acceptor, phosphine activation may be broadly applicable to complexes exhibiting a mismatch between hard (high oxidation state/charge) acceptors and soft phosphine donors. Indeed phosphines are considered poor donors for hard acceptors and coordination to such centres generally requires enforcement by chelate or pincer ligands.^11–13^

**Chart 1:**
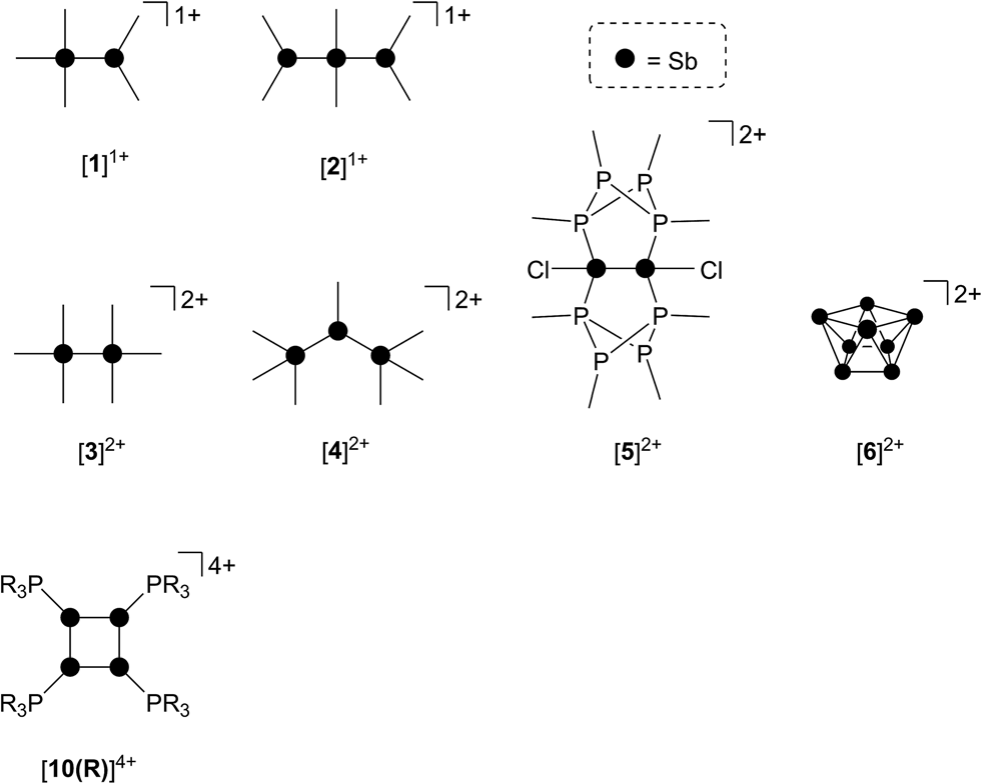
Structurally confirmed cations featuring Sb–Sb bonds. See text for references.

As part of a systematic evolution of p-block element phosphine complexes, we have sought derivatives featuring multiply-charged, hard acceptors and now report evidence of a new phosphine ligand activation pathway in the coordination sphere of polycationic Sb^III^ centres. Specifically, reductive elimination of diphosphonium dications (Scheme 1e) from trialkylphosphine complexes of Sb^III^ has been demonstrated, comprehensively defining a fundamental P–P bond forming redox process. The reduction products are the unusual *cyclo*-tetra(stibinophosphonium) tetracations [**10(R)**]^4+^, representing a new *catena*-homocyclic framework.^14^ Examples of cationic homocycles for p-block metalloids are limited to unsupported selenium and tellurium dications^15^ and heavily substituted silicon^16^ or germanium^17^ monocations. For antimony, a number of acyclic catenated monocations ([**1**]^1+^ and [**2**]^1+^)^18–20^ and dications ([**3**]^2+^, [**4**]^2+^, [**5**]^2+^ and [**6**]^2+^)^19,21–23^ have recently been isolated (Chart 1), but generally on small scales, precluding further reactivity studies of these interesting species. Enabled by a rational and large scale synthetic protocol for cations [**10(R)**]^4+^, we now report the reaction chemistry of the prototypical derivative, [**10(Me)**]^4+^, debuting the coordination chemistry of a new *catena*-element framework.

## Results and discussion

### Reactions of PR_3_ with FSb(OTf)_2_ and Sb(OTf)_3_

**Scheme 2:**
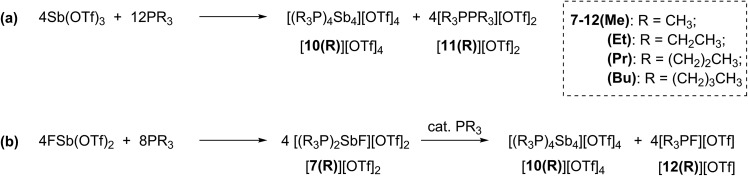
Formations of cations [**10(R)**]^4+^, [**11(R)**]^2+^, and [**12(R)**]^1+^ as triflate salts in reaction mixtures containing trialkylphosphines and (a) Sb(OTf)_3_ or (b) FSb(OTf)_2_.

Combinations of FSb(OTf)_2_ or Sb(OTf)_3_ with PR_3_ (R = Me, Et, Pr, or Bu) in MeCN solvent at the optimized stoichiometries given in Scheme 2 have been investigated. The ^31^P, ^13^C, ^19^F and ^1^H NMR spectra of reaction mixtures indicate quantitative formation of *cyclo*-tetra(stibinophosphonium) triflate salts [**10(R)**][OTf]_4_ (R = Me, Et, Pr, Bu) together with derivatives of [**11(R)**][OTf]_2_ (Scheme 2a) or [**12(R)**][OTf] (Scheme 2b). Large lattice enthalpy differences permit separation of the monocationic salts [**12(R)**][OTf] from the tetracationic salts [**10(R)**][OTf]_4_ by fractional crystallization, whereas pure salts cannot be isolated from mixtures of dicationic [**11(R)**][OTf]_2_ and [**10(R)**][OTf]_4_.

**Fig. 1:**
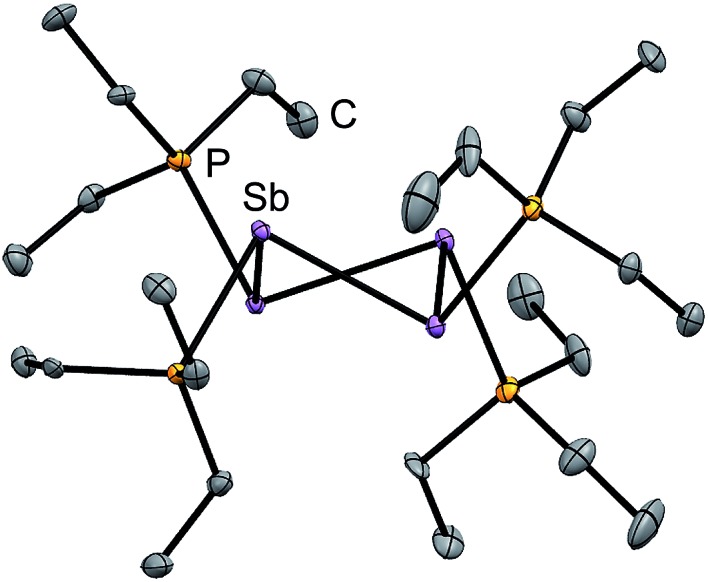
Solid-state molecular structure of the cation in [**10(Et)**][OTf]_4_·(MeCN). Hydrogen atoms, anions and solvent molecules have been omitted for clarity. Thermal ellipsoids are drawn at 30% probability level. Metric parameters are given in Table 1.

Four derivatives of [**10(R)**][OTf]_4_ (R = Me, Et, Pr, Bu) have been characterized spectroscopically by solution NMR spectroscopy, and two derivatives, [**10(Me)**][OTf]_4_ and [**10(Et)**][OTf]_4_, comprehensively characterized. The solid-state structures of these two salts have been determined by X-ray crystallography to confirm formulae involving a tetracation with a folded Sb_4_-cyclic core with four exocyclic PR_3_ units and four triflate anions (Fig. 1 and Table 1). The Sb–Sb bond lengths are very similar for [**10(Me)**]^4+^ [2.8354(6)–2.8797(5) Å] and [**10(Et)**]^4+^ [2.838(2)–2.884(2) Å] and their values are marginally longer than those observed in rare examples of *catena*-antimony cations [*cf.* [**1**]^1+^ = 2.8205(12) Å,^18^ 2.8278(3) Å,^19^ [**2**]^1+^ = 2.8203(4) Å,^20^ [**3**]^2+^ = 2.7624(11) Å and 2.7867(12) Å,^21^ [**4**]^2+^ = 2.811(1) Å and 2.830(1) Å,^19^ and [**5**]^2+^ = 2.8484(12) Å and 2.8353(12) Å ^22^]. Consistent with the high Lewis acidity of the Sb_4_ core, the P–Sb distances [2.552(2)–2.578(2) Å] are similar to those observed in other triflate salts such as [(Me_3_P)_2_SbCl][OTf]_2_ [2.5950(4) Å and 2.5834(4) Å] and [(Me_3_P)SbPh_2_][OTf] [2.5584(4) Å].^32^

**Table 1:** Selected bond lengths (Å) and angles (°) in the solid-state structures of [**10(Me)**][OTf]_4_·(MeCN)_3_ (ref. 26) and [**10(Et)**][OTf]_4_·MeCN

	[**10(Me)**][OTf]_4_·(MeCN)_3_	[**10(Et)**][OTf]_4_·(MeCN)
*d*(Sb–Sb)	2.8354(6)–2.8797(5)	2.838(2)–2.884(2)
*d*(Sb···Sb)	3.7471(5)–3.7792(5)	3.646(2)–3.804(2)
*d*(P–Sb)	2.552(2)–2.564(2)	2.553(3)–2.578(2)
*d*_min_(Sb···O_OTf_)	3.210(4)	2.871(8)
<(Sb–Sb–Sb)	82.09(2)–83.36(2)	78.62(3)–82.88(4)
<(P–Sb–Sb)	93.74(4)–99.60(4)	91.91(7)–98.68(7)
<(Sb–Sb–Sb–Sb)	38.85(2)–39.27(2)	43.30(2)–44.56(3)

A number of Sb–O_OTf_ contacts are also observed, the shortest of which measure 3.210(4) Å for [**10(Me)**]^4+^ and 2.871(8) Å for [**10(Et)**]^4+^. Given the high molecular charge, these values are expectedly smaller than the sum of the van der Waals radii (∑_r,vdW_ = 3.61 Å)^24^ but nevertheless significantly longer than the sum of the single bond covalent radii (∑_r,cov_ = 2.05 Å)^25^ for the two elements. For [**10(Me)**]^4+^, a gas-phase optimization^26^ of the cation at the MP2 level in the absence of the triflate anions produced a geometry that is essentially identically to that observed experimentally, and we therefore infer that the anion contacts do not distort the structural features to a measurable extent.

**Scheme 3:**
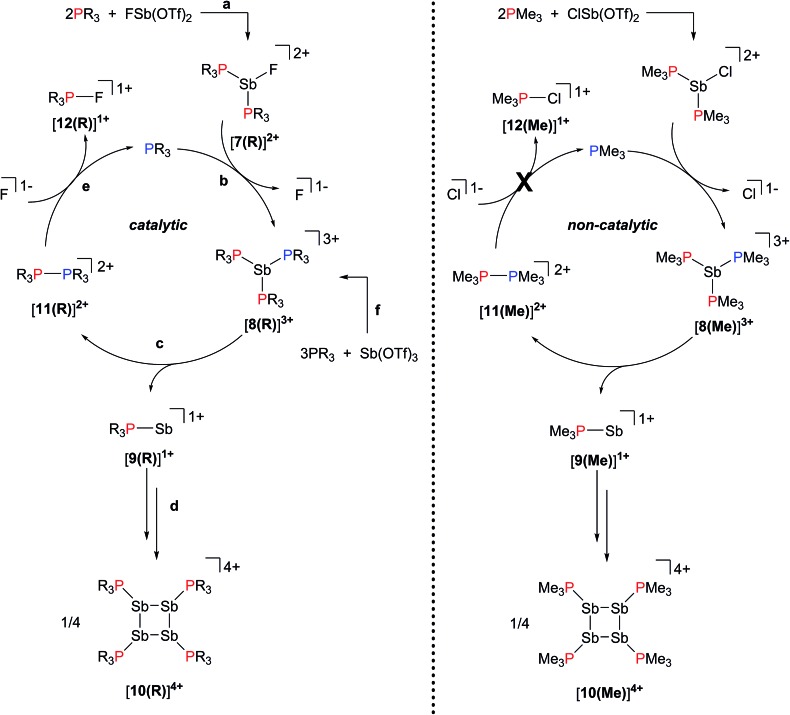
(Left) Proposed catalytic mechanism for the formation of derivatives of cations [**7(R)**]^2+^, [**8(R)**]^3+^, [**9(R)**]^1+^, [**10(R)**]^4+^, [**11(R)**]^2+^, and [**12(R)**]^1+^. See text for descriptions of **a–f**. (Right) Non-catalytic formation of [**10(Me)**]^4+^ from the reaction of [(Me_3_P)_2_SbCl]^2+^ with PMe_3_.

The reactions in Scheme 2a represent a two electron reduction of each antimony(iii) center and collectively, an eight electron reductive coupling of four antimony centers to form derivatives of [**10(R)**]^4+^. In Scheme 2a, eight of the twelve equivalents of phosphine are involved in the redox process, being oxidatively coupled to give four diphosphonium cations, [**11(R)**]^2+^,^27,28,31^ and the remaining four equivalents represent ligands on the reduced antimony(i) centers of [**10(R)**]^4+^. Scheme 2b describes a similar redox process that involves formation of [**11(R)**]^2+^ as transients, which are converted to the corresponding fluorophosphonium cations, [**12(R)**]^1+^, in the presence of the fluoride ion, as envisaged in the mechanism outlined in Scheme 3 (left). The key feature in both processes is reductive elimination of a diphosphonium unit from a hard, tricationic Sb^III^ centre to give a soft, monocationic Sb^I^ centre, representing a novel mode of phosphine ligand activation in the coordination sphere of metals (Scheme 1e).

**Fig. 2:**
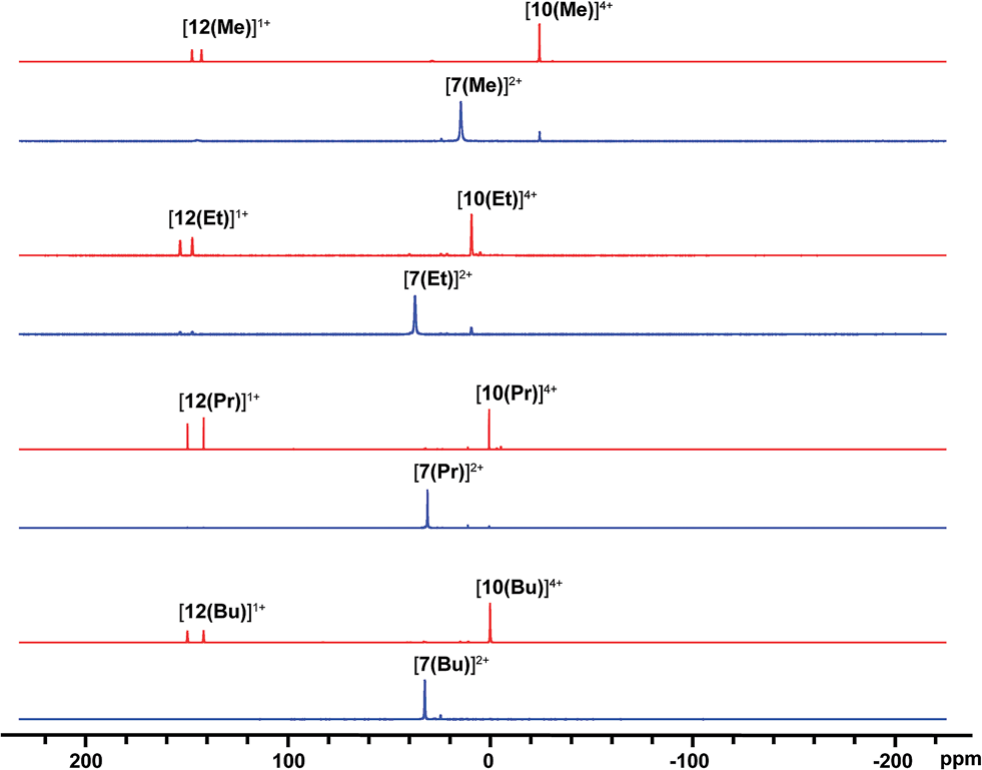
^31^P{^1^H} NMR spectra (202.5 MHz, 298 K, CD_3_CN) of reaction mixtures containing FSb(OTf)_2_ and PR_3_ leading to the formation of [**7(R)**]^2+^ (blue) and, upon addition of 15 mol% PR_3_, to [**12(R)**]^1+^, and [**10(R)**]^4+^ (red). See Table 2 for chemical shift data.

^31^P NMR spectra (Fig. 2) of reaction mixtures containing PR_3_ and FSb(OTf)_2_ in a 2 : 1 stoichiometry show a broad doublet in the +20 to +40 ppm range and the signal due to the free phosphine (–60 to –20 ppm) is not observed. The ^19^F NMR spectra of these mixtures show a broad triplet in the range –170 to –175 ppm and no evidence of FSb(OTf)_2_. The broadness of peaks in the ^31^P and ^19^F NMR spectra is consistent with the connectivity of these nuclides to a quadrupolar antimony center [*I* = 5/2 for ^121^Sb (57%), 7/2 for ^123^Sb (43%)],^40^ and we assign these signals to the dicationic *bis*-phosphine cations [**7(R)**]^2+^, which are stable as MeCN solutions (Scheme 3a). Upon addition of *ca*. 5 mol% of phosphine to these solutions, the ^31^P NMR signals due to cations [**7(R)**]^2+^ are replaced over 16 hours by doublets corresponding to [**12(R)**]^1+^ (*δ* ^31^P: +140 to +150 ppm, ^1^ *J*_PF_ = 950–1000 Hz) and a singlet in the –25 to 0 ppm range, corresponding to [**10(R)**]^4+^. Addition of *ca*. 15 mol% of phosphine increases the rate of the reaction and effects complete conversion of [**7(R)**]^2+^ to [**12(R)**]^1+^ and [**10(R)**]^4+^ within an hour.

We propose that displacement of fluoride from [**7(R)**]^2+^ by added phosphine yields the highly-charged trications [**8(R)**]^3+^ (Scheme 3b), which undergo reductive elimination of [**11(R)**]^2+^ and [**9**]^1+^ (Scheme 3c). Subsequent tetramerization of the six-valence electron cations [**9(R)**]^1+^ to [**10(R)**]^4+^ (Scheme 3d), and displacement of PR_3_ from [**11(R)**]^2+^ by fluoride gives [**12(R)**]^1+^, regenerating the phosphine catalyst (Scheme 3e). Cyclization of transients [**9(R)**]^1+^ is analogous to the formation of tetrameric (Mes-E)_4_ (Mes = 2,4,6-trimethylphenyl, E = As or Sb) *via* catalytic extrusion of Mes-As^I^ from a zirconium complex^29^ or Mes-Sb^I^ from a hafnium complex.^30^ Nucleophilic displacement of PMe_3_ has been reported^31^ in reaction mixtures of [**11(Me)**][ClO_4_]_2_ and [NEt_4_][F], and we have further confirmed that the equimolar reaction of [**11(Me)**][OTf]_2_ with CsF (Fig. S1, ESI†) yields a 1 : 1 mixture of PMe_3_ and [**12(Me)**]^1+^. Trications [**8(R)**]^3+^ are also implicated in the formation of [**10(R)**]^4+^ from Sb(OTf)_3_ (Scheme 3f) and we have previously reported^26^ the structure of the ternary salt [**8(Me)**][**11(Me)**][OTf]_5_ from a 1 : 3 mixture of Sb(OTf)_3_ and PMe_3_ at –30 °C (*vide infra*). Consistent with the role of [**8(Me)**]^3+^ as an intermediate, the same reaction stoichiometry yields only [**10(Me)**][OTf]_4_ and [**11(Me)**][OTf]_2_ at ambient temperature.

The ^31^P NMR spectra of reaction mixtures containing [(Me_3_P)_2_SbCl]^2+^ and 20 mol% PMe_3_ show only partial conversion to [**10(Me)**][OTf]_4_ and [**11(Me)**][OTf]_2_ after 48 hours. Additionally, a broad signal at +10.4 ppm is also observed (Fig. S2, ESI†), which is close to the average for the values in [(Me_3_P)_2_SbCl]^2+^ (+15.8 ppm) and [(Me_3_P)_2_SbCl_2_]^1+^ (+6.2 ppm),^32^ suggesting that the free chloride ion is sequestered in an equilibrium between the starting material and [(Me_3_P)_2_SbCl_2_]^1+^. Consequently, nucleophilic attack by chloride to liberate free phosphine from [**11(Me)**]^2+^ is precluded in these reaction mixtures and neither [Me_3_PCl]^1+^ nor free phosphine are detected by ^31^P NMR spectroscopy.

Signifying the role of free phosphine as a catalyst, formation of [**10(Me)**]^4+^ does not occur catalytically in the chloride system because the reaction is arrested upon formation of [**11(Me)**]^2+^, which is the spectroscopically detected oxidation product. Generation of free phosphine from diphosphonium, the turnover limiting step, does not take place (Scheme 3, right). In contrast, no diphosphonium is detected in reactions involving the fluoroantimony complexes [**7(R)**]^2+^ (Scheme 3, left), where, due to nucleophilic attack by fluoride anions on [**11(R)**]^2+^, only the fluorophosphoniums [**12(R)**]^1+^ are detected as the oxidation product and the formation of [**10(R)**]^4+^ occurs catalytically in the presence of free PR_3_. Differences in the reactivity of homologous Sb–X (X = Cl, F) complexes towards Lewis acids have been noted previously.^33^

Solution NMR data for derivatives of [**7(R)**]^2+^, [**8(R)**]^3+^, [**10(R)**]^4+^, [**11(R)**]^2+^, and [**12(R)**]^1+^ are summarized in Table 2, with evidence for the assignments discussed below. It has not been possible to detect or isolate derivatives of [**9(R)**]^1+^. Attempts to trap these cations, or radical intermediates arising from one-electron processes, in the presence of a twenty-fold excess of 2,3-dimethyl-1,3-butadiene were unsuccessful.

**Table 2:** Solution NMR data (CD_3_CN, 298 K) for derivatives of [**7(R)**]^2+^, [**8(R)**]^3+^, [**10(R)**]^4+^, [**11(R)**]^2+^, and [**13**]^2+^. Values in parentheses indicate literature values for chemical shifts of known compounds. Values in square brackets denote peak width at half-maximum where the expected ^2^ *J*_PF_ coupling was not observed (n.o.)

	*δ* ^31^P (ppm)	*δ* ^19^F (ppm)	^*n*^ *J*_PF_ (Hz)
[**7(Me)**]^2+^	+15.9	–178.2	44
[**7(Et)**]^2+^	+38.0 [23]	–174.2 [73]	n.o.
[**7(Pr)**]^2+^	+29.3	–173.4	41
[**7(Bu)**]^2+^	+32.4 [62]	–173.9 [52]	n.o.
[**13**]^2+^	+41.3	–187.1	39
[**8(Me)**]^3+^	+21.3	—	—
[**8(Et)**]^3+^	+27.8	—	—
[**8(Pr)**]^3+^	+22.1	—	—
[**8(Bu)**]^3+^	+22.4	—	—
[**10(Me)**]^4+^	–24.4	—	—
[**10(Et)**]^4+^	+9.3	—	—
[**10(Pr)**]^4+^	+0.6	—	—
[**10(Bu)**]^4+^	+0.5	—	—
[**11(Me)**]^2+^	+28.5 (28.4)^27^	—	—
[**11(Et)**]^2+^	+38.5 (21)^31^	—	—
[**11(Pr)**]^2+^	+31.4 (32)^31^	—	—
[**11(Bu)**]^2+^	+31.0 (32)^31^	—	—
[**12(Me)**]^1+^	+148.0	–138.0	948
[**12(Et)**]^1+^	+150.2	–159.3	973
[**12(Pr)**]^1+^	+145.6	–154.7	971
[**12(Bu)**]^1+^	+145.6	–155.4	971

Derivatives of [**7(R)**]^2+^ represent the first examples of phosphine complexes of fluoroantimony acceptors although numerous fluoroantimony complexes with hard, oxidatively-resistant donors such as pyridines,^33,34^ ethers,^35–38^ and pnictogen oxides^34,39^ have been reported. The ^2^ *J*_PF_ couplings for [**7(Me)**]^2+^ and [**7(Pr)**]^2+^ are resolved as a doublet in the ^31^P NMR spectra and as a triplet in the ^19^F NMR spectra, consistent with an AX_2_ spin system. Fine structure could not be resolved for [**7(Et)**]^2+^ and [**7(Bu)**]^2+^ even under the dilute conditions and low temperature (–30 °C) employed to mitigate broadening due to exchange.

Although [**7(Me)**][OTf]_2_ and [**7(Et)**][OTf]_2_ have both been isolated as analytically pure substances and spectroscopically characterized, we were unable to obtain X-ray quality crystals. Moreover, to the best of our knowledge, there are no known examples of ^2^ *J*_PF_ coupling constants through an antimony centre for direct comparison with our assigned NMR data. For this reason, we prepared and isolated the analogous [(*dmpe*)SbF][OTf]_2_, [**13**][OTf]_2_, from an equimolar mixture of 1,2-*bis*(dimethylphosphino)ethane (*dmpe*) and FSb(OTf)_2_ in MeCN. The solid state structure of [**13**][OTf]_2_, as determined by X-ray crystallography, shows a dimeric arrangement with the cations bridged by O–S–O contacts from the triflate anions, and additional interactions with two non-bridging triflate anions, as shown in Fig. S3 (ESI).† The pyramidal geometry at Sb in the cation is retained in solution, as demonstrated by the two non-equivalent methyl group resonances in the ^13^C (6.1 and 7.2 ppm) and ^1^H NMR (1.86 and 2.10 ppm) spectra. Crucially, the expected ^2^ *J*_PF_ coupling was unambiguously observed (Fig. S4, ESI†) in signals due to [**13**]^2+^, and the chemical shift and coupling constants are comparable to those assigned to derivatives of [**7(R)**]^2+^ (Table 2).

It was not possible to isolate salts of [**8(R)**]^3+^ due to their high reactivity, consistent with their disproportionation to [**11(R)**]^2+^ and [**10(R)**]^4+^ in solution as proposed above. The ^31^P NMR signals assigned to derivatives of [**8(R)**]^3+^ are singlets and broadened (Δ*ν*_1/2_ = 90–500 Hz), presumably due to a combination of the quadrupolar antimony nuclides^40^ and dynamic ligand exchange. Nevertheless, [**8(Me)**][OTf]_3_ has been detected as a co-crystallate with [**11(Me)**][OTf]_2_ in a 3 : 1 reaction mixture of PMe_3_ and Sb(OTf)_3_ at –30 °C.^26^ The molecular structure of [**8(Me)**]^3+^ (Fig. S5, ESI†) in the salt [**8(Me)**][**11(Me)**][OTf]_5_ shows a pyramidal arrangement around the Sb atom with three P–Sb lengths in the range 2.5974(8)–2.6115(7) Å and P–Sb–P angles in the range 101.33(3)–102.40(2)°. In addition, three interion Sb–O contacts are observed in the 2.791(2)–2.960(2) Å range (*cf.* ∑_r,vdW_ = 3.61 Å),^24^ with each contact appearing *trans* to a P–Sb bond, illustrating a triple displacement of triflate anions from Sb(OTf)_3_ by three PMe_3_ ligands.

**Scheme 4:**
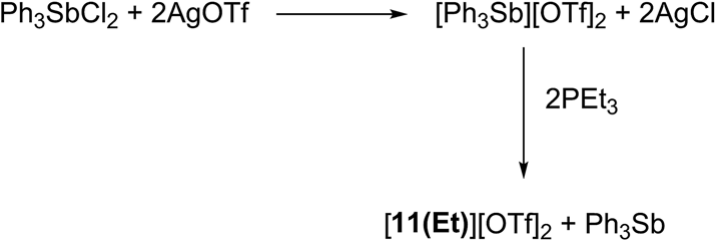
Synthesis of [**11(Et)**][OTf]_2_ *via* oxidative coupling of PEt_3_ with [Ph_3_Sb][OTf]_2_.

**Fig. 3:**
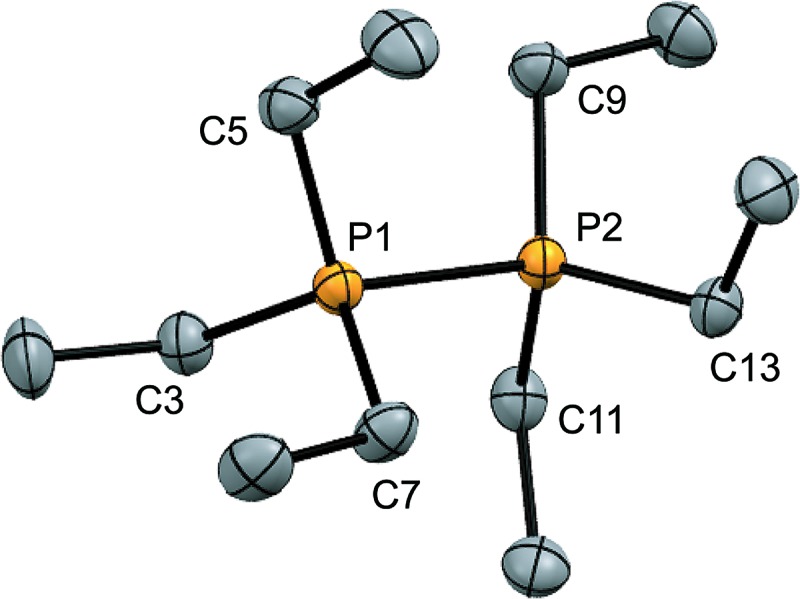
Molecular structure of the cation in [**11(Et)**][OTf]_2_ in the solid state. Hydrogen atoms and triflate anions have been omitted for clarity. Thermal ellipsoids are drawn at 30% probability level. Bond lengths (Å) and angles (°) are as follows: P1–P2 = 2.2209(8), P1–C3 = 1.800(2), P1–C5 = 1.802(2), P1–C7 = 1.806(2), P2–C9 = 1.796(2), P2–C11 = 1.809(2), P2–C13 = 1.798(2), C3–P1–P2–C9 = 28.7 (1).

Signals attributed to derivatives of [**11(R)**]^2+^ are assigned by comparison with previously reported ^31^P chemical shifts for their triflate or perchlorate salts in MeCN for [**11(Me)**]^2+^, [**11(Pr)**]^2+^, and [**11(Bu)**]^2+^.^27,31^ Isolation of [**11(Pr)**][OTf]_2_ enabled comprehensive characterization, including X-ray structural determination and we have reported this data elsewhere.^41^ The salt [**11(Et)**][OTf]_2_ has been prepared independently from a 2 : 1 reaction of PEt_3_ with *in situ* generated Ph_3_Sb(OTf)_2_, according to Scheme 4,^42^ and the structure of the cation is shown in Fig. 3. The P–P bond length [2.2209(8) Å] is comparable to that in rare examples of acyclic diphosphonium dications such as [**11(Me)**]^2+^ [2.198(2) Å]^27^ or [Me_3_PPEt_3_]^2+^ [2.216(1) Å],^27^ and a partially eclipsed conformation is observed between the six ethyl groups. In contrast to a previously assigned ^31^P NMR chemical shift (+21 ppm)^31^ for [**11(Et)**][ClO_4_]_2_, which was not structurally authenticated, [**11(Et)**][OTf]_2_ exhibits a ^31^P NMR chemical shift of +38.5 ppm.

**Fig. 4:**
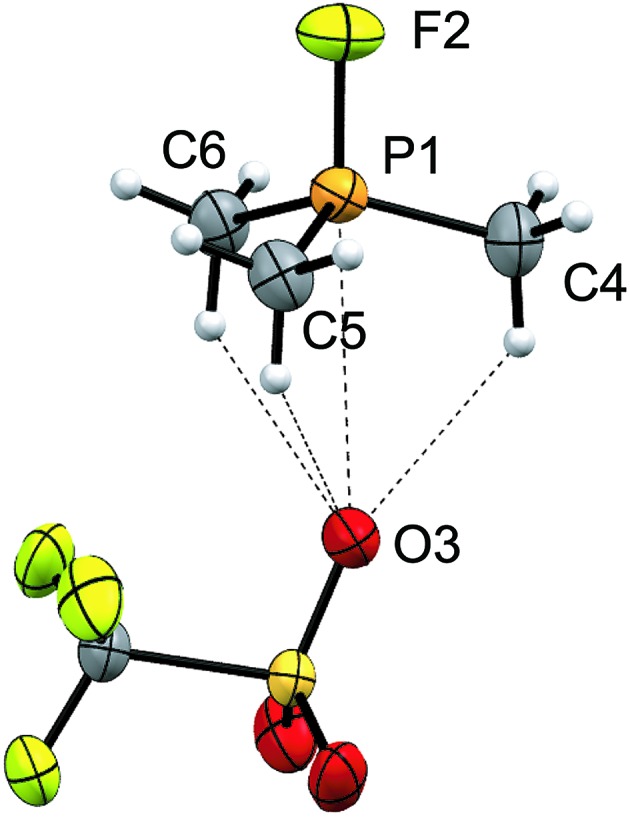
Molecular structure of [**12(Me)**][OTf] in the solid state. Thermal ellipsoids are drawn at 30% probability level. Bond lengths (Å) and angles (°) are as follows: P1–F2 = 1.551 (1), P1–C = 1.757(2), 1.755 (2), 1.755 (2), P1–O3 = 3.301(2), F2–P1–O3 = 177.34(8), F2–P1–C = 106.38(6), 105.2(1), 106.38(6).

The ^31^P and ^19^F NMR resonances attributed to fluorophosphonium cations [**12(R)**]^1+^ were confirmed by comparison to literature values or independent synthesis of triflate salts from small-scale equimolar mixtures of the appropriate phosphine with XeF_2_ followed by treatment with one equivalent of TMSOTf. To the best of our knowledge, the solid-state structure of [**12(Me)**][OTf] (Fig. 4) represents the first structural characterization of a trialkylfluorophosphonium salt, and involves three hydrogen bonds with the triflate anion in addition to one weak contact [3.301(2) Å] between a triflate oxygen atom and the phosphorus atom, which is marginally shorter than Σ_r,vdw_ for the two elements (3.320 Å).^24^ The O–P–F angle generated by this contact is 177.34(8)°, representing adjustment of the *D*_3h_ structure of Me_3_PF_2_.^43^

The ^31^P NMR chemical shifts for species in Table 2 over a 150 ppm range but within each class of cations, generally decrease in the order *δ*(R = Et) > *δ*(R = Pr) ≈ *δ*(R = Bu) > *δ*(R = Me). Notably, the chemical shifts of the free phosphines (range of 30 ppm) also show the same order, providing additional support for the proposed assignments (Fig. S6, ESI†).

Attempts to isolate cations [**7(R)**]^2+^ or [**10(R)**]^4+^ with bulky phosphines such as P^i^Pr_3_ were unsuccessful. A ^31^P NMR assay of the reaction mixture containing P^i^Pr_3_ and FSb(OTf)_2_ in a 2 : 1 ratio displayed numerous fluorine containing products as indicated by the observation of spin system with P–F couplings but no pure compounds could be isolated. A 3 : 1 mixture of P^i^Pr_3_ with Sb(OTf)_3_ also gave a complex mixture of products at room temperature which could not be separated. Deprotonation of MeCN solvent was observed upon refluxing the reaction mixture for short periods or stirring at room temperature for 16 hours. We conclude that steric bulk at the α-carbon of the phosphine hinders the coordination required for clean transformation of *bis*-phosphine cations [**7(R)**]^2+^ to *tris*-phosphine cations [**8(R)**]^3+^.

While the initial isolation of [**10(Me)**][OTf]_4_ as a pure substance was achieved on a 150 mg scale (*ca.* 0.1 mmol), making it unamenable to reactivity studies, reaction conditions have now been optimized for a one-pot, three-step reaction (ESI) to give reproducible yields of analytically pure [**10(Me)**][OTf]_4_ and [**10(Et)**][OTf]_4_ on a scale up to 10 g. Consistent with the exquisite sensitivity of these compounds towards hydrolysis and oxidation, particularly in solution, the key determinant of purity and reactions yields is the rigorous drying and deoxygenation of the solvent and careful application of dynamic vacuum (*ca.* 10^–1^ mbar) in the latter stages of the reaction to avoid free phosphine-catalyzed decomposition (*vide infra*).

### Thermolysis and photolysis of [**10(Me)**][OTf]_4_

The four-membered ring of [**10(Me)**]^4+^ contains four of the six Sb–Sb bonds required to make neutral, tetrahedral Sb_4_, which is directly analogous to P_4_ and As_4_. Moreover, [**10(Me)**]^4+^ also contains four phosphine ligands which may be susceptible to further reductive elimination of two diphosphonium dications, [**11(Me)**]^2+^, to yield neutral Sb_4_. While P_4_ and As_4_ are well characterized, Sb_4_ has not been isolated as a bulk solid, and only one solid-state structural determination has been made using a scanning tunnelling microscope to characterize a thin film of Sb_4_ under ultra-high-vacuum conditions.^44^ In this context, we envisioned the thermal or photochemical decomposition of [**10(Me)**][OTf]_4_ as a route to bulk solid Sb_4_.

**Scheme 5:**
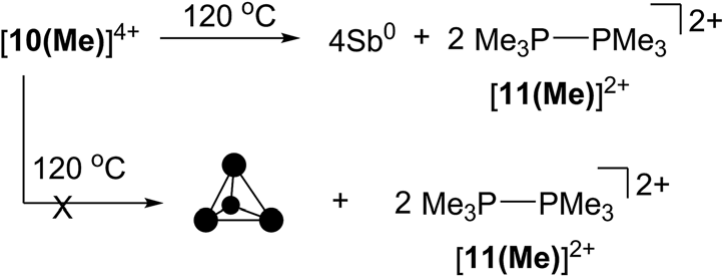
Thermolysis of [**10(Me)**][OTf]_4_ to yield [**11(Me)**][OTf]_2_ and elemental antimony.

A sample of solid [**10(Me)**][OTf]_4_ (yellow-colored) heated under argon at 120 °C for 16 hours turned black, consistent with the formation of elemental antimony (Scheme 5). A CD_3_CN extract of the black product showed ^31^P, ^1^H and ^13^C NMR signals corresponding exclusively to [**11(Me)**]^2+^ as the sole oxidation product. A Raman spectrum of the black solid (Fig. S7, ESI†) matched that of the amorphous α-phase (110 cm^–1^, 150 cm^–1^)^45^ of antimony rather than the reported Raman spectrum of tetrahedral Sb_4_ in argon matrix (138 cm^–1^, 179 cm^–1^, 242 cm^–1^).^46^ Identical results were obtained when heating was carried out in the dark, under vacuum, or in solution (toluene). Irradiating solid [**10(Me)**][OTf]_4_ or as a solution in MeCN at 256 nm for 3 hours at room temperature had no measurable effect. It should be noted that in the gas phase tetrahedral Sb_4_ is the preferred allotrope of the element up to 1050 K.^47^ It is possible that despite its gaseous stability, tetrahedral Sb_4_ is thermodynamically unstable with respect to its amorphous phases in the condensed state, preventing its isolation as a solid and is, in this context, analogous to tetrahedral As_4_ (yellow arsenic) which spontaneously decomposes to a hexagonal allotrope (grey arsenic, α-As) at room temperature.^48^

The thermolysis described above must be carried out in rigorously dried glassware, the surface of which has been treated with Me_3_SiCl to silanize terminal –OH groups. Samples heated without prior passivation of glassware produced elemental antimony and [**11(Me)**][OTf]_2_, but also showed resonances due to [Me_3_PH]^1+^ and a singlet at +115.6 ppm in the ^31^P{^1^H} NMR spectrum (CD_3_CN) of the reaction mixture, consistent with formation of [Me_3_POPMe_3_]^2+^. This assignment is supported by an independent synthesis from a 2 : 1 mixture of Me_3_PO and triflic anhydride, using a well-established protocol for these reagents.^49^ We interpret the formation of these by-products as being due to the reaction of the extremely moisture sensitive [**10(Me)**][OTf]_4_ with surface hydroxyl groups in nonsilanized glassware.

### Reactions of [**10(Me)**][OTf]_4_ with R_*n*_PX_(3–*n*)_; X = H, Cl; *n* = 1, 2

**Scheme 6:**
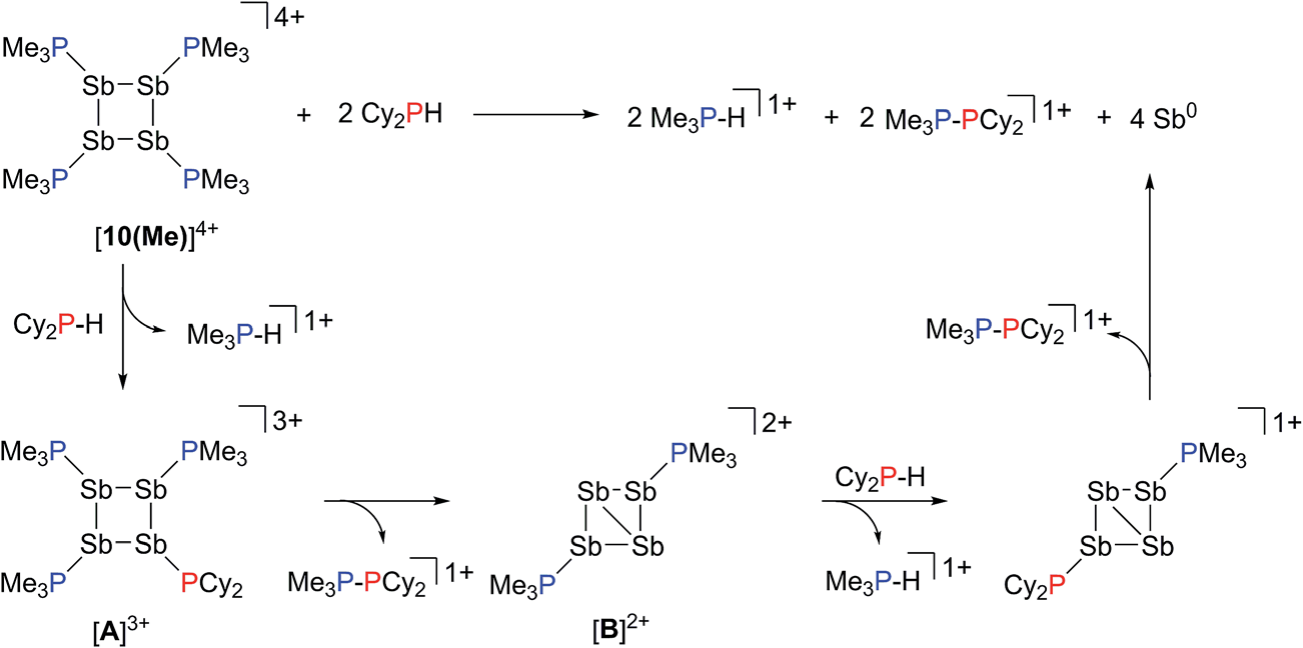
Formation of [Me_3_PH]^1+^ and [Me_3_PPCy_2_]^1+^ from the reaction of [**10(Me)**][OTf]_4_ with Cy_2_PH.

Addition of a solution of R_2_PH (R = C_6_H_11_ (Cy), Ph) to a clear yellow-colored solution of [**10(Me)**][OTf]_4_ in MeCN results in immediate deposition of a fine black precipitate and loss of the yellow coloration. The ^31^P{^1^H} NMR spectra of reaction supernatants show a singlet due to [Me_3_PH]^1+^ and two doublets characteristic of phosphinophosphonium cations [Me_3_PPR_2_]^1+^ (R = Cy, Ph) with typical ^1^ *J*_PP_ values in the 300–350 Hz range (Scheme 6).^50^ Cation [Me_3_PPPh_2_]^1+^ is known^51^ and the assignment of [Me_3_PPCy_2_]^1+^ was confirmed by comparison of chemical shifts and coupling constants with literature values^50^ for phosphinophosphonium salts and by elemental analysis.

**Scheme 7:**
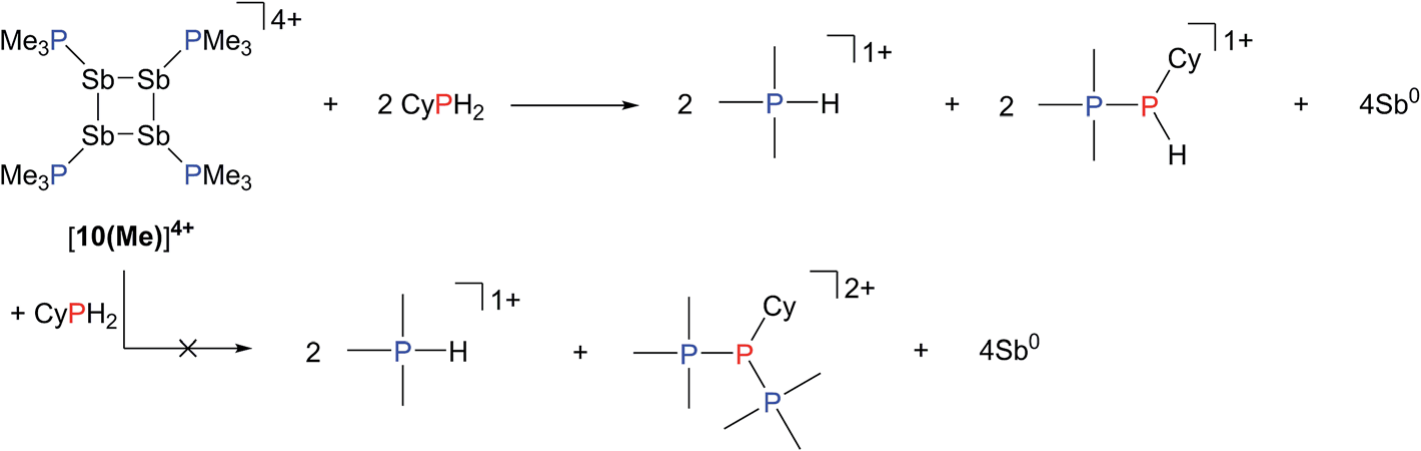
Reaction of CyPH_2_ with [**10(Me)**][OTf]_4_.

**Fig. 5:**
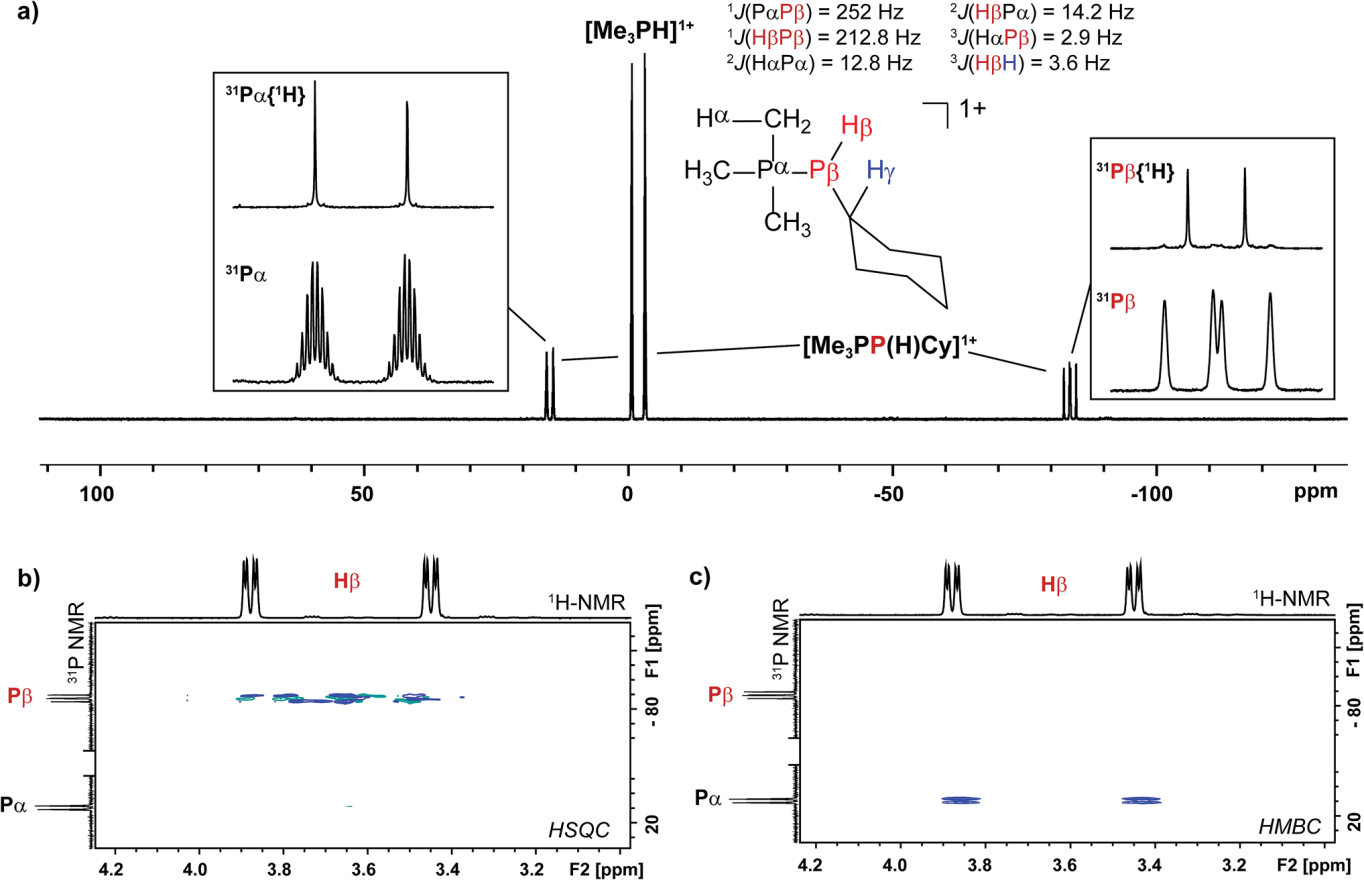
(a) ^31^P NMR spectrum of the crude reaction mixture containing CyPH_2_ and [**10(Me)**][OTf]_4_ in a 2 : 1 ratio. Insets show detailed views of the ^31^P{^1^H} and ^31^P NMR resonances assigned to the Me_3_P- (left) and -P(H)Cy (right) fragments in [Me_3_PP(H)Cy]^1+^. A list of coupling constants deduced from the combination of ^31^P and ^1^H NMR is also given. (b) Sections of the ^31^P/^1^H HSQC spectrum showing a ^1^ *J*_PH_ coupling between P_β_ and H_β_. (c) Sections of the ^31^P/^1^H HMBC spectrum showing a ^2^ *J*_PH_ coupling between P_α_ and H_β_.

Analogously, addition of a solution of RPH_2_ (R = Cy, ^*t*^butyl) to a solution of [**10(Me)**][OTf]_4_ results in immediate precipitation of elemental antimony. The ^31^P NMR spectra of these reaction mixtures show complete consumption of RPH_2_ and [**10(Me)**][OTf]_4_, and formation of a singlet due to [Me_3_PH]^1+^ and a pair of doublets assigned to [Me_3_PP(H)R]^1+^ (Scheme 7). Consistent with this formulation, the ^31^P–^1^H coupled NMR spectrum of the reaction involving CyPH_2_ shows (Fig. 5a) both ^1^ *J*_PP_ and ^1^ *J*_HP_ couplings for the phosphinic signal centered at –83.6 ppm. The P_α_–P_β_(H_β_)Cy connectivity is also confirmed in the ^1^H NMR spectrum of the reaction mixture (Fig. S8, ESI†), where H_β_ resonates at +3.65 ppm exhibiting ^1^ *J*_HβPβ_, ^2^ *J*_HβPα_, and ^3^ *J*_HβHγ_ couplings, the last of these arising from coupling to the *ipso* proton (H_γ_) of the cyclohexyl ring. The methyl protons (H_α_) around P_α_ also show the expected ^2^ *J*_HαPα_ and ^3^ *J*_HαPβ_ couplings, indicating a P–P bond. Finally, a two-dimensional ^31^P/^1^H HSQC (Fig. 5b) spectrum, which was optimized to show one-bond couplings, shows coupling between H_β_ and P_β_ but no coupling involving H_β_ and P_α_. The corollary two-dimensional HMBC experiment (Fig. 5c), optimized to exclude one-bond couplings, shows coupling between H_β_ and P_α_, but no coupling involving H_β_ and P_β_. Despite numerous attempts, it was not possible to separate [Me_3_PP(H)Cy][OTf] from [Me_3_PH][OTf], precluding elemental analysis or structural determination by X-ray diffraction. Nevertheless, to the best of our knowledge this is the first spectroscopic detection of an H-phosphinophosphonium cation.

The formation of [Me_3_PH]^1+^ and the phosphinophosphonium salts is understood in broad terms as a metathesis step followed by a reductive elimination step as outlined in Scheme 6. We speculate that coordination of Cy_2_PH to one of the antimony centres in [**10(Me)**][OTf]_4_ is followed by intramolecular deprotonation by PMe_3_ to yield the observed [Me_3_PH]^1+^ cation and a tricationic intermediate, [**A**]^3+^. This trication can undergo rapid intramolecular reductive elimination of the first equivalent of the phosphinophosphonium cation to give dication [**B**]^2+^. A second round of coordination, deprotonation and reductive elimination completes the reduction of antimony to its elemental form and furnishes the observed distribution of products. Unfortunately, the partially reduced species were not observed and appear to be fleeting intermediates. Nevertheless, formation of [Me_3_PP(H)R]^1+^ from reactions involving primary phosphines (Scheme 7) is consistent with the proposed mechanism, although it is unclear why the second deprotonation does not occur to yield the corresponding dication [(Me_3_P)_2_PR]^2+^. As before, Raman analysis of the black precipitate matches the amorphous α-phase of metallic antimony rather than pyramidal Sb_4_.

**Scheme 8:**
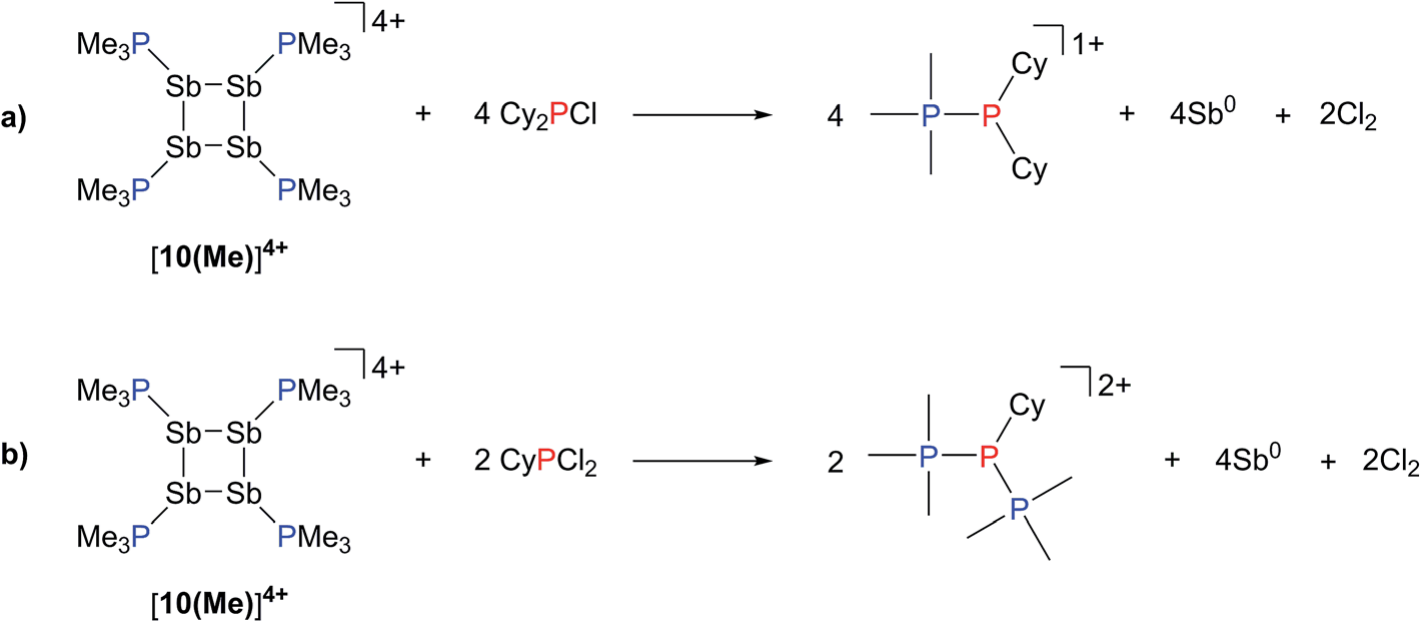
Formation of [Me_3_PPCy_2_]^1+^, [Me_3_PP(Cl)Cy]^1+^, and [(Me_3_P)_2_PCy]^2+^ from the reaction of [**10(Me)**][OTf]_4_ with Cy_2_PCl or CyPCl_2_.

The observation that [**10(Me)**][OTf]_4_ serves as a source of PMe_3_, which deprotonates added primary and secondary phosphines, implies a labile and polarized P–Sb bond that undergoes facile heterolytic cleavage. Consistently, addition of Cy_2_PCl or CyPCl_2_ to a solution of [**10(Me)**][OTf]_4_ results in quantitative formation of [Me_3_PPCy_2_]^1+^ or [(Me_3_P)_2_PCy]^2+^,^52^ respectively, concomitant with deposition of elemental antimony (Scheme 8). In these cases, [**10(Me)**]^4+^ behaves overall as a chloride abstractor and phosphine donor. We tentatively propose formation of chloroantimony species as transients that undergo loss of chlorine gas to yield elemental antimony as there is no evidence of Sb–Cl bond stretching modes in the Raman spectra of the insoluble black solid isolated from these reactions. However, since no products expected from reactions of dissolved Cl_2_ could be detected, the fate of the chlorine atoms cannot yet be definitively described. When intermediate stoichiometries of CyPCl_2_ are employed, formation of the known [Me_3_PP(Cl)Cy]^1+^ cation^53^ is also observed, indicating a single chloride abstraction event, that is analogous to the formation of [Me_3_PP(H)Cy]^1+^ in reactions with CyPH_2_. ^31^P NMR data for [Me_3_PPCy_2_][OTf], [Me_3_PP(H)Cy][OTf], [(Me_3_P)_2_PCy][OTf]_2_, and [Me_3_PP(Cl)Cy][OTf] are given in Table 3.

**Table 3:** ^31^P NMR (CD_3_CN, 298 K) chemical shifts and coupling constants for products obtained from the reaction of Cy_2_PH, CyPH_2_, Cy_2_PCl, and CyPCl_2_ with [**10(Me)**][OTf]_4_

	^31^P (ppm)	^1^ *J*_PP_ (Hz)	Reference
[Me_3_PPCy_2_][OTf]	+12.8, –5.1	327	This work
[Me_3_PP(H)Cy][OTf]^*a*^	+14.8, –83.6	252	This work
[(Me_3_P)_2_PCy][OTf]_2_	+22.7, –30.8	307, 326	52
[Me_3_PP(Cl)Cy][OTf]	+23.0, +78.4	326	53

^*a*^ ^1^ *J*_PH_ = 214 Hz.

### Reaction of [**10(Me)**][OTf]_4_ with PMe_3_

**Scheme 9:**
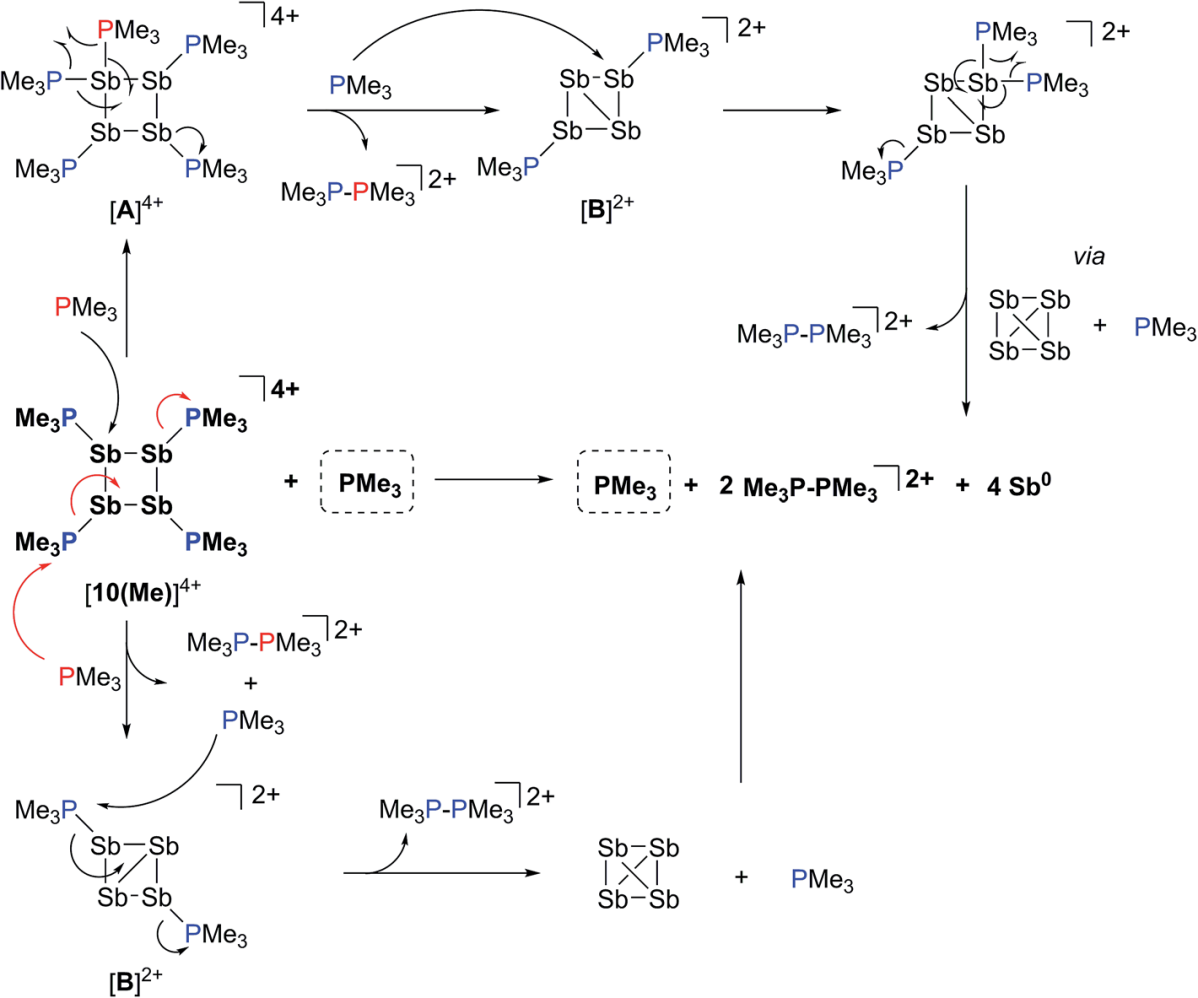
Catalytic decomposition of [**10(Me)**][OTf]_4_ by PMe_3_ *via* nucleophilic attack at Sb (upper half) or P (lower half).

The ^31^P NMR spectrum of a reaction mixture containing 15 mol% of PMe_3_ and [**10(Me)**][OTf]_4_ shows slow disappearance of the signal due to the latter and evolution of broadened signals due to [**11(Me)**]^2+^ and free PMe_3_. Concomitantly, a mirror of antimony is deposited in the reaction vessel. Within 12 hours at 298 K, there is no evidence of [**10(Me)**]^4+^, while signals due to [**11(Me)**]^2+^ and free PMe_3_ persist, consistent with complete decomposition of the tetracation, catalyzed by PMe_3_. The proposed mechanisms (Scheme 9) involve nucleophilic attack by the added phosphine at either the antimony or the phosphorus centres. Attack at a stibine should yield intermediate [**A**]^4+^ (Scheme 9), featuring a hypercoordinate antimony centre. Several examples of such hypervalent P–Sb complexes have been reported.^32,3^ Due to its high charge concentration, this complex is predicted to be strongly oxidizing, and, in a process analogous to reductive elimination from [**8(Me)**]^3+^, an equivalent each of [**11(Me)**]^2+^ and intermediate [**B**]^2+^ (Scheme 9) can be generated, enabling dissociation of PMe_3_. Alternatively, attack at one of the phosphorus centres of [**10(Me)**]^4+^ directly generates intermediate [**B**]^2+^ together with [**11(Me)**]^2+^ and PMe_3_. The liberated phosphine can further reduce [**B**]^2+^ by a second nucleophilic attack either at Sb or P to evolve the second equivalent of [**11(Me)**]^2+^ and yield fully reduced antimony. Nucleophilic attack by a neutral two-electron ligand at tetracoordinate trimethylchlorophosphonium, trimethylphosphonium and dimethylthiophosphonium cations has been demonstrated previously.^54–56^ The broadness of signals for [**11(Me)**]^2+^ and PMe_3_ in these reaction mixtures is attributed to an exchange process that is also detected when free PMe_3_ is added to a solution of [**11(Me)**][OTf]_2_.

The catalytic decomposition of [**10(Me)**][OTf]_4_ in the presence of PMe_3_ explains the difficulties encountered during synthesis of this salt. For instance, if the addition rate of PMe_3_ to FSb(OTf)_2_ is too high, a dark orange solution is obtained which rapidly deposits elemental antimony (see note in Experimental section). However, if a dynamic vacuum is applied to the dark orange solution to remove the volatile PMe_3_ (b.p. = 38 °C), the solution maintains a yellow colour, leading to the formation of [**10(Me)**][OTf]_4_. Moreover reactions with Lewis bases that displace PMe_3_ must be carried out with explicit steps to remove the liberated phosphine in order to avoid decomposition (*vide infra*).

### Reaction of [**10(Me)**][OTf]_4_ with [Li][*nacnac* ^(dipp)^]

**Scheme 10:**
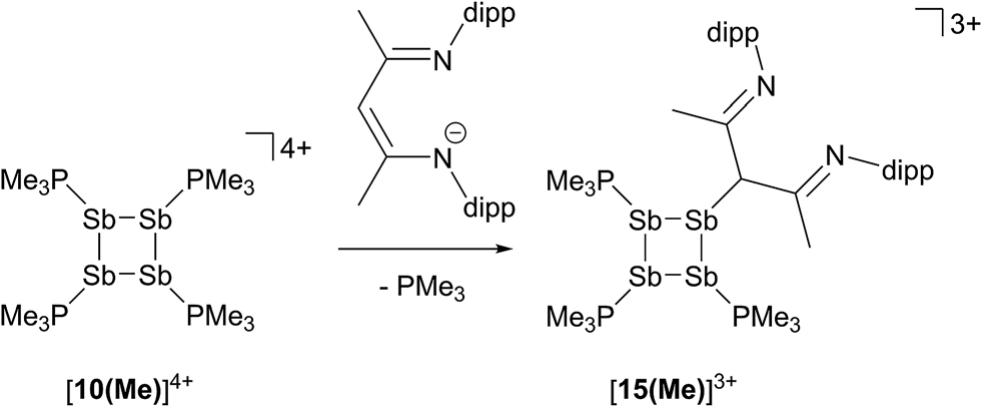
Formation of [**15(Me)**]^3+^ by nucleophilic displacement of PMe_3_ from [**10(Me)**]^4+^.

**Fig. 6:**
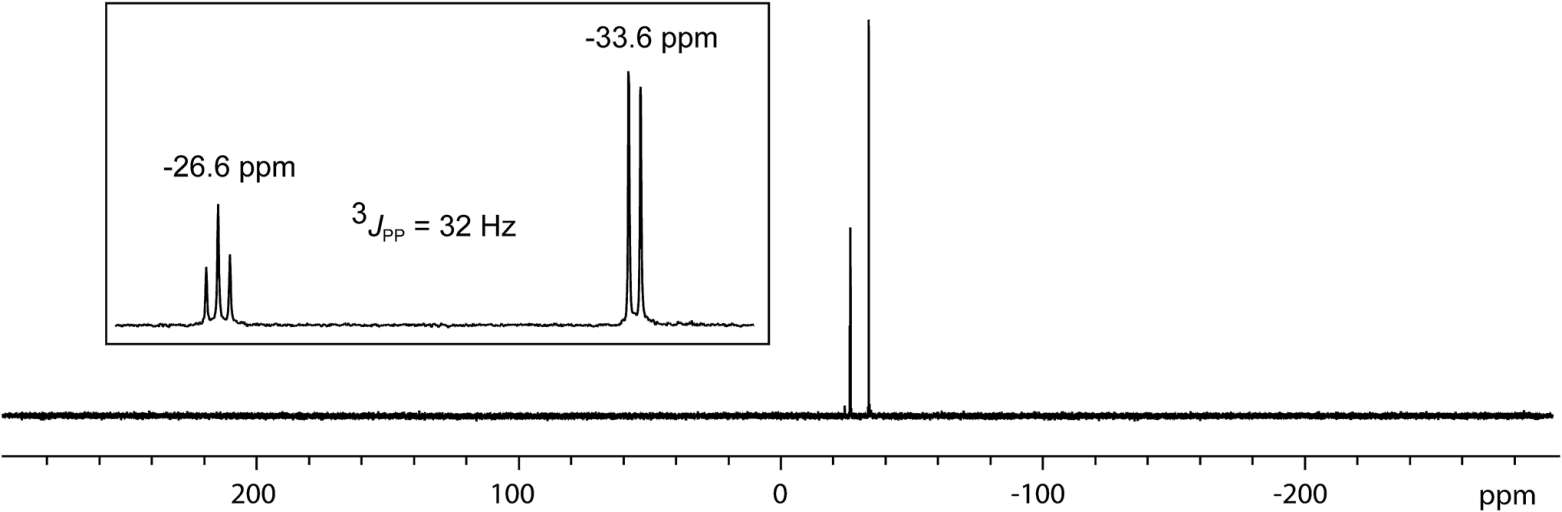
^31^P{^1^H} NMR spectrum of [**15(Me)**][OTf]_3_ at 298 K in CD_3_CN. Inset shows the AX_2_ spin system due to ^3^ *J*_PP_ coupling between two equivalent and one unique phosphorus environment in [**15(Me)**]^3+^.

In contrast to the sterically unhindered and neutral base PMe_3_, a bulky and anionic base is expected to yield products arising from ligand substitution rather than from addition. Consistently, the ^31^P{^1^H} NMR spectra of equimolar reaction mixtures of [**10(Me)**][OTf]_4_ and Li[*nacnac* ^(dipp)^] (dipp = 2,6-diisopropylphenyl), indicate quantitative formation of [**15(Me)**][OTf]_3_ (Scheme 10). The 1,3-diketiminate anion [*nacnac* ^(dipp)^]^1–^, abbreviated as *nacnac*, displaces one PMe_3_ ligand from [**10(Me)**]^4+^ to give [(Me_3_P)_3_Sb_4_(*nacnac*)]^3+^ ([**15(Me)**]^3+^), which is an analogue of [(Me_3_P)_3_Sb_4_(PCy_2_)]^3+^ (intermediate [**A**]^3+^ in Scheme 6). The ^31^P{^1^H} NMR spectrum (Fig. 6) of [**15(Me)**][OTf]_3_ shows the expected AX_2_ spin system [–26.6 ppm (*triplet*), –33.6 ppm (*doublet*), ^3^ *J*_PP_ = 32 Hz] and a corresponding AX_2_ spin system [–6.3 ppm (*triplet*), –2.5 ppm (*doublet*), ^3^ *J*_PP_ = 23 Hz] is also observed for [**15(Et)**]^3+^, prepared from the reaction of [**10(Et)**]^4+^ with [Li][*nacnac* ^(dipp)^]. Isolation of [**15(Me)**][OTf]_3_ is only possible when the reaction is performed under a mild dynamic vacuum to remove the displaced phosphine, which effects redox decomposition at high concentrations, presumably *via* similar mechanisms as described above for [**10(Me)**]^4+^.

**Fig. 7:**
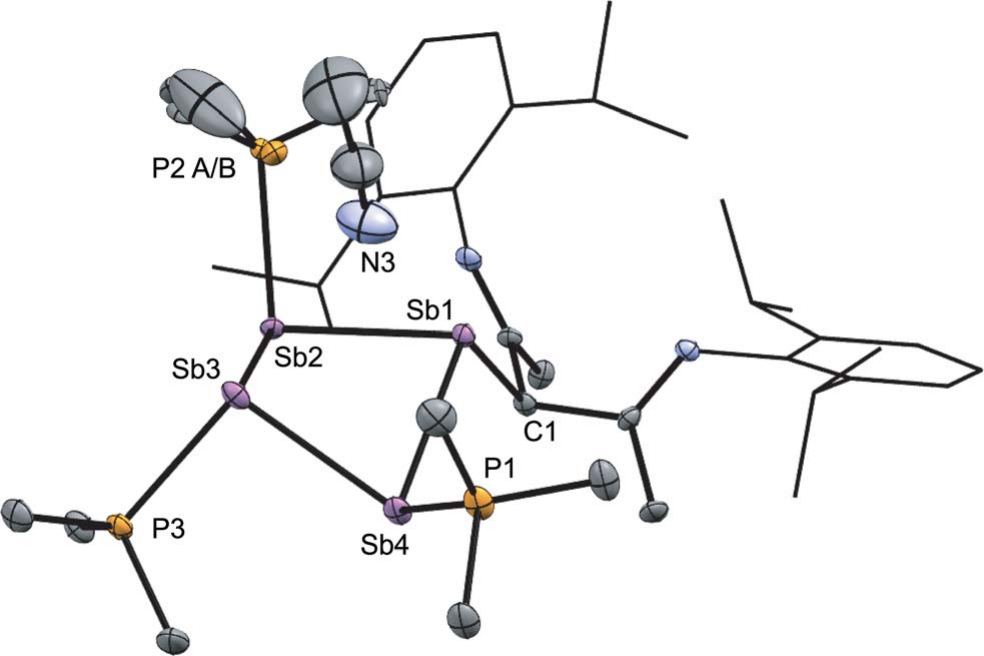
Molecular structure of the cation in [**15(Me)**][OTf]_3_·MeCN in the solid state. Hydrogen atoms and triflate anions have been omitted for clarity. Thermal ellipsoids are drawn at 30% probability level. Bond lengths (Å) and angles (°) are as follows: Sb1–Sb2 = 2.8209(5), Sb2–Sb3 = 2.8457(5), Sb3–Sb4 = 2.8501(5), Sb1–Sb4 = 2.8612(5), P1–Sb4 = 2.538(2), P2A–Sb2 = 2.548(5), P2B–Sb2 = 2.604(9), P3–Sb3 = 2.541(1), C1–Sb1 = 2.209(5), Sb1–Sb3 = 3.7344(5), Sb2–Sb4 = 3.7003(5), Sb1–N3 = 3.42(1), Sb3–N3 = 3.19(1), Sb1–Sb2–Sb3 = 82.45(1), Sb2–Sb3–Sb4 = 81.03(1), Sb3–Sb4–Sb1 = 81.67(2), Sb4–Sb1–Sb2 = 81.26(1), Sb1–Sb2–Sb3–Sb4 = –42.10(2).

The solid-state structure of the cation in [**15(Me)**][OTf]_3_·MeCN (Fig. 7) shows three phosphine ligands and the rare γ-coordination mode for the *nacnac* substituent,^57^ which, to the best of our knowledge, has not been observed for haloantimony centres bound to this substituent.^58^ Heteroleptic substitution is very rare in antimony homocycles^59^ and examples for cationic systems have not been reported. The range of Sb–Sb [2.8209(5)–2.8612(5) Å] and Sb–P [2.538(5)–2.604(9) Å] distances are similar to those in [**10(Me)**]^4+^ indicating minimal distortion of the Sb_4_ ring upon displacement of PMe_3_ with *nacnac*. While [**15(Me)**][OTf]_3_ is stable in the solid state under inert atmosphere, ^31^P{^1^H} NMR spectra of MeCN solutions show decomposition over five days at 20 °C to elemental antimony, [**10(Me)**][OTf]_4_ and [**11(Me)**][OTf]_2_ (Fig. S9, ESI†).

To assess whether or not bonding *via* the γ carbon of *nacnac* is a general feature of antimony compounds and because *nacnac* functionalized antimony centers are rare in the literature, we also prepared (*nacnac*)Sb(OTf)_2_ by salt metathesis between an equimolar mixture of *in situ* generated Sb(OTf)_3_ and [Li][*nacnac* ^(dipp)^]. Upon removal of LiOTf, the compound was isolated as a pure substance and comprehensively characterized. The molecular structure of (*nacnac*)Sb(OTf)_2_, determined by X-ray diffraction, shows a see-saw geometry around antimony with two strongly-interacting triflate anions in axial positions (Fig. S10, ESI†). In contrast to γ-coordination observed for [**15(Me)**]^3+^, *N*,*N*′-chelation is observed for (*nacnac*)Sb(OTf)_2_, and we attribute the difference in bonding modes to the different steric environments around antimony in the two compounds, rather than intrinsic features of the *nacnac*-Sb interaction.

Interestingly, the ^19^F resonances for the two triflate CF_3_ groups in (*nacnac*)Sb(OTf)_2_ are different (–78.3 and –78.4 ppm), implying a rigid ring system with non-equivalent positions above and below the plane of the ring. Consistently, the isopropyl substituents show two unique resonances for the C_ipso_ protons. Furthermore, there is restricted rotation around the C_ipso_–C_phenyl_ bond giving rise to four unique signals for the methyl groups in the ^1^H NMR spectrum of the compound. We speculate that this is due to solution-phase persistence of the weak hydrogen bonding interactions between the nitrogen atoms and the isopropyl C_ipso_ protons, detected as short contacts in the solid state molecular structure (Fig. S10, ESI†).

### Reaction of [**10(Me)**][OTf]_4_ with *dmap*

**Fig. 8:**
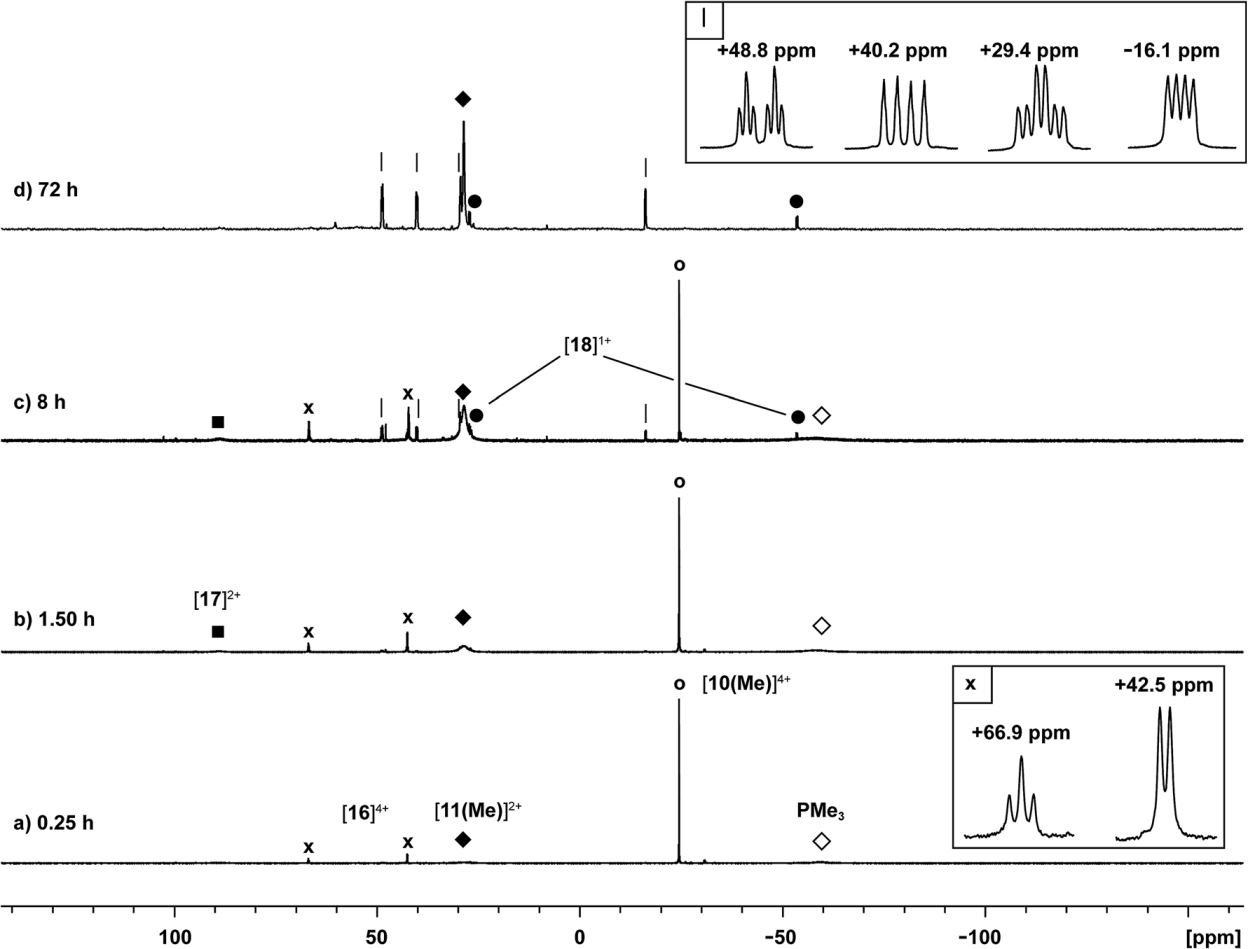
Formation of [**11(Me)**]^2+^ (♦), [**16**]^4+^ (×), [**17**]^2+^ (■), [**18**]^1+^ (), and PMe_3_ () in the equimolar reaction of *dmap* with [**10(Me)**][OTf]_4_ (○). Peaks labelled with a vertical line (|) correspond to an unidentified product. Insets show the spin systems observed for [**16**]^4+^ (×) and the unidentified product (|).

**Scheme 11:**
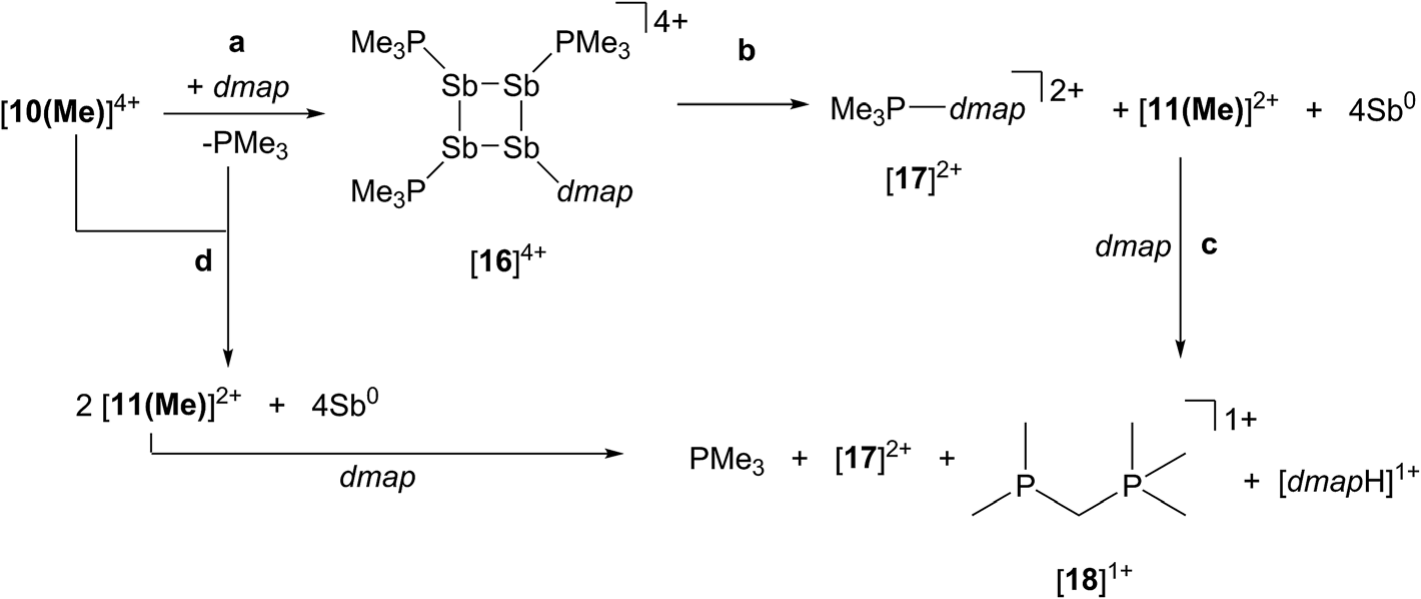
Proposed pathways to formation of [**16**]^4+^ (a), [**17**]^2+^, [**11(Me)**]^2+^, elemental antimony (b and d), and [**18**]^1+^ (c) in reaction mixtures containing [**10(Me)**][OTf]_4_ and *dmap* in a 1 : 1 stoichiometry.

**Scheme 12:**
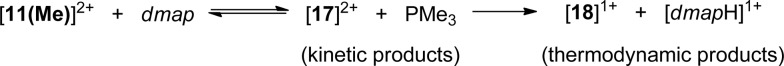
Kinetic and thermodynamic pathways in the reaction between [**11(Me)**]^2+^ and *dmap*.

The reaction of [**10(Me)**][OTf]_4_ with 4-dimethylaminopyridine (*dmap*) has been examined by ^31^P NMR (Fig. 8) and shows displacement of one phosphine ligand by *dmap* (Scheme 11). It was not possible to isolate the resulting products. Following filtration of the reaction mixture (black suspension), the yellow-green filtrate shows the expected AX_2_ spin system (triplet at +66.9 ppm, doublet at +42.5 ppm, ^3^ *J*_PP_ = 24 Hz), tentatively assigned to [(Me_3_P)_3_Sb_4_(*dmap*)]^4+^ ([**16**]^4+^), and broad signals due to PMe_3_ (–62 ppm), [Me_3_P(*dmap*)]^2+^ ([**17**]^2+^, +89.0 ppm)^54^ and [**11(Me)**]^2+^. Within hours, signals due to [Me_3_PCH_2_PMe_2_]^1+^ ([**18**]^1+^, doublet at –53.9 ppm, doublet at +26.0 ppm, ^2^ *J*_PP_ = 58 Hz)^60^ appear in the ^31^P NMR spectrum and a significant amount of [*dmap*H]^+^ is observed by ^1^H NMR spectroscopy. We propose that the latter two species arise from deprotonation of the slightly acidic protons of [**11(Me)**]^2+^ by *dmap* and the subsequent rearrangement of [Me_3_PPMe_2_CH_2_]^1+^ (Scheme 11). Consistently, a 1 : 1 control reaction of *dmap* and [**11(Me)**]^2+^ initially shows broad signals for [**17**]^2+^ and free PMe_3_ as the kinetic products, but within 4 hours signals due to [**18**]^1+^ and [*dmap*H]^1+^ are observed, revealing them to be the thermodynamic products (Scheme 12, Fig. S11 in ESI†). In addition to [**18**]^1+^, four unique and mutually coupled phosphorus environments (by ^31^P NMR spectroscopy) are also observed which could not been assigned definitively.

The ^3^ *J*_PP_ coupling constant for the signal assigned to [**16**]^4+^ (24 Hz) is comparable to the values in [**15(Me)**]^3+^ (32 Hz) and [**15(Et)**]^3+^ (23 Hz). However, the ^31^P{^1^H} NMR chemical shifts observed for [**16**]^4+^ (A: +66.9, X_2_: +42.5) are significantly downfield from those of [**15(Me)**]^3+^ and [**15(Et)**]^3+^ (Table 4), and this cannot be attributed solely to the different formal charges in the species as the PMe_3_ groups in tetracationic [**10(Me)**]^4+^ resonate at –24.5 ppm. We propose that *dmap*-stabilized main-group cations generally show ^31^P NMR chemical shifts that are substantially downfield from their PMe_3_-stabilized homologues (Table 4) due to the greater electronegativity of nitrogen relative to phosphorus, supporting the assignment for [**16**]^4+^.

**Table 4:** Comparison of ^31^P NMR chemical shifts for some phosphorus containing main-group cations stabilized by PMe_3_ or *dmap*. Values for tetracoordinate phosphorus centers are given in parentheses, where applicable

	^31^P NMR	Reference
[Me_2_PPMe_3_]^1+^	+18 (–59)	27
[Me_2_P(*dmap*)]^1+^	+91	54
[Ph_2_P(PMe_3_)]^1+^	–23 (+15)	51
[Ph_2_P(*dmap*)]^1+^	+88	55
[Me_2_(S)PPMe_3_]^1+^	+16 (+38)	56
[Me_2_(S)P(*dmap*)]^1+^	+88	56
[**11(Me)**]^2+^	(+28.4)	27
[**17**]^2+^	(+89.0)	54
[**10(Me)**]^4+^	(–24.5)	This work
[**15(Me)**]^3+^	(–26.6), (–33.6)	This work
[**15(Et)**]^3+^	(–2.5), (–6.3)	This work
[**16**]^4+^	(+66.9), (+42.5)	This work
[**18**]^1+^	(+26.0), –53.9	60

### Reaction of [**10(Me)**][OTf]_4_ with [**10(Et)**][OTf]_4_

Neutral *catena*-antimony rings are known to participate in ring–ring equilibria unless bulky substituents or dilute solutions are employed. For instance, solutions of hexaphenylcyclohexastibine (Ph_6_Sb_6_) equilibrate to give a mixture of four-, five-, and six-membered rings suggesting labile Sb–Sb bonds.^61^

**Fig. 9:**
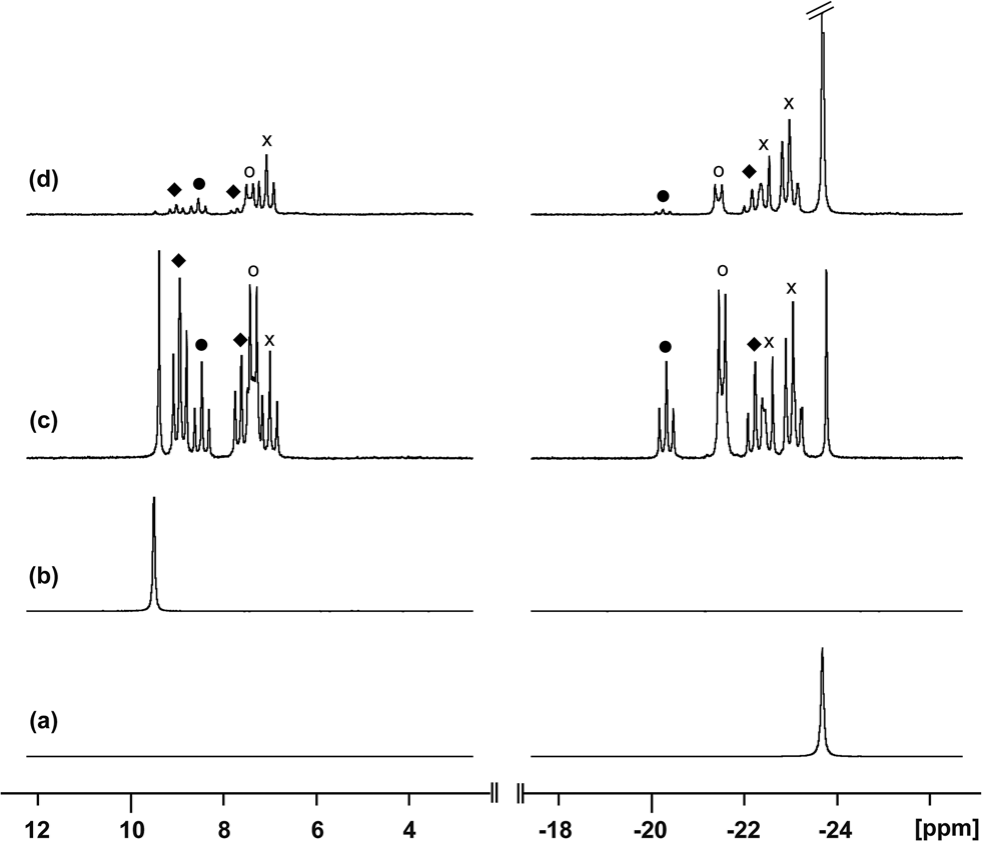
^31^P{^1^H} NMR spectra (CD_3_CN, 298 K) of (a) [**10(Me)**]^4+^, (b) [**10(Et)**]^4+^, (c) 1 : 1 mixture of [**10(Me)**]^4+^ and [**10(Et)**]^4+^, and (d) 1 : 1 mixture of [**10(Me)**]^4+^ and PEt_3_. Symbols denote tentative assignments for [**10(Me)_3_(Et)**]^4+^ (×), *cis*-[**10(Me)_2_(Et)_2_**]^4+^ (○), *trans*-[**10(Me)_2_(Et)_2_**]^4+^ (), and [**10(Me) (Et)_3_**]^4+^ (♦).

**Scheme 13:**
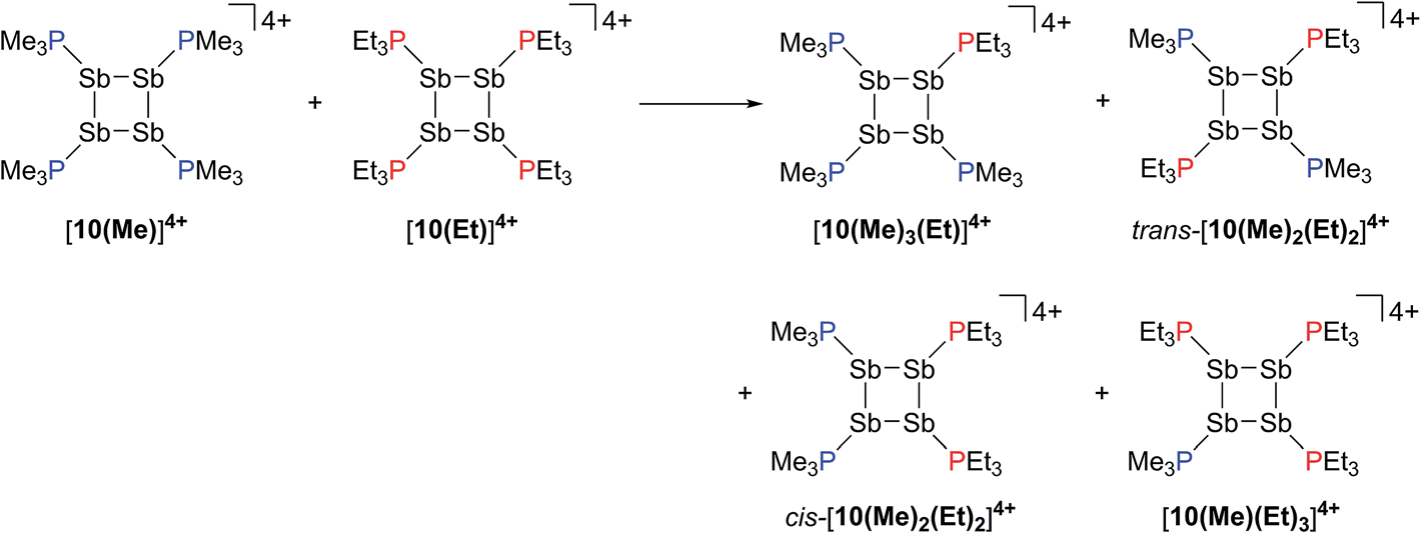
Proposed formation of constitutional isomers from the equimolar reaction of [**10(Me)**]^4+^ and [**10(Et)**]^4+^.

To assess the possibility of preparing heteroleptic derivatives of [**10(R)**][OTf]_4_, pure samples of [**10(Me)**][OTf]_4_ and [**10(Et)**][OTf]_4_ were combined in a 1 : 1 stoichiometry. The ^31^P{^1^H} NMR spectrum (Fig. 9c) of the resulting mixture suggests formation of multiple constitutional isomers of [(PMe_3_)_*x*_(PEt_3_)_(4–*x*)_Sb_4_]^4+^, implicating a scrambling process in the two ring systems *via* Sb–Sb or P–Sb bond cleavage. A scrambling process involving Sb–Sb cleavage has been described previously for distibines.^62^ However, a control experiment, where free PEt_3_ was added to [**10(Me)**][OTf]_4_, also showed (Fig. 9d) formation of these isomers. Therefore a nucleophilic displacement pathway, where a bound PR_3_ ligand is displaced by an added PR′3 ligand, cannot be precluded. However this displacement route to heteroleptically substituted derivatives also yields significant amounts of [**11(Me)**]^2+^ and elemental antimony, presumably due to free PMe_3_ catalyzed decomposition of [**10(Me)**][OTf]_4_ as described earlier. Although, it has not yet been possible to purify these reaction mixtures and isolate the first examples of heteroleptically substituted *catena*-antimony rings, signal multiplicities consistent with AM_2_X, A_2_X_2_, and AA′XX′ spin systems are observed, as expected from a mixture of [**10(Me)_3_(Et)**]^4+^, *cis*/*trans*-[**10(Me)_2_(Et)_2_**]^4+^, and [**10(Me)(Et)_3_**]^4+^ (Scheme 13). Moreover, the coupling constants lie in the 21–26 Hz range and are comparable to ^3^ *J*_PP_ coupling constants detected in [**15(Me)**]^3+^ (32 Hz), [**15(Et)**]^3+^ (23 Hz), and [**16**]^4+^ (24 Hz). Collectively, these data enable a tentative assignment of the spectral features observed in Fig. 9.

## Conclusions

**Scheme 14:**
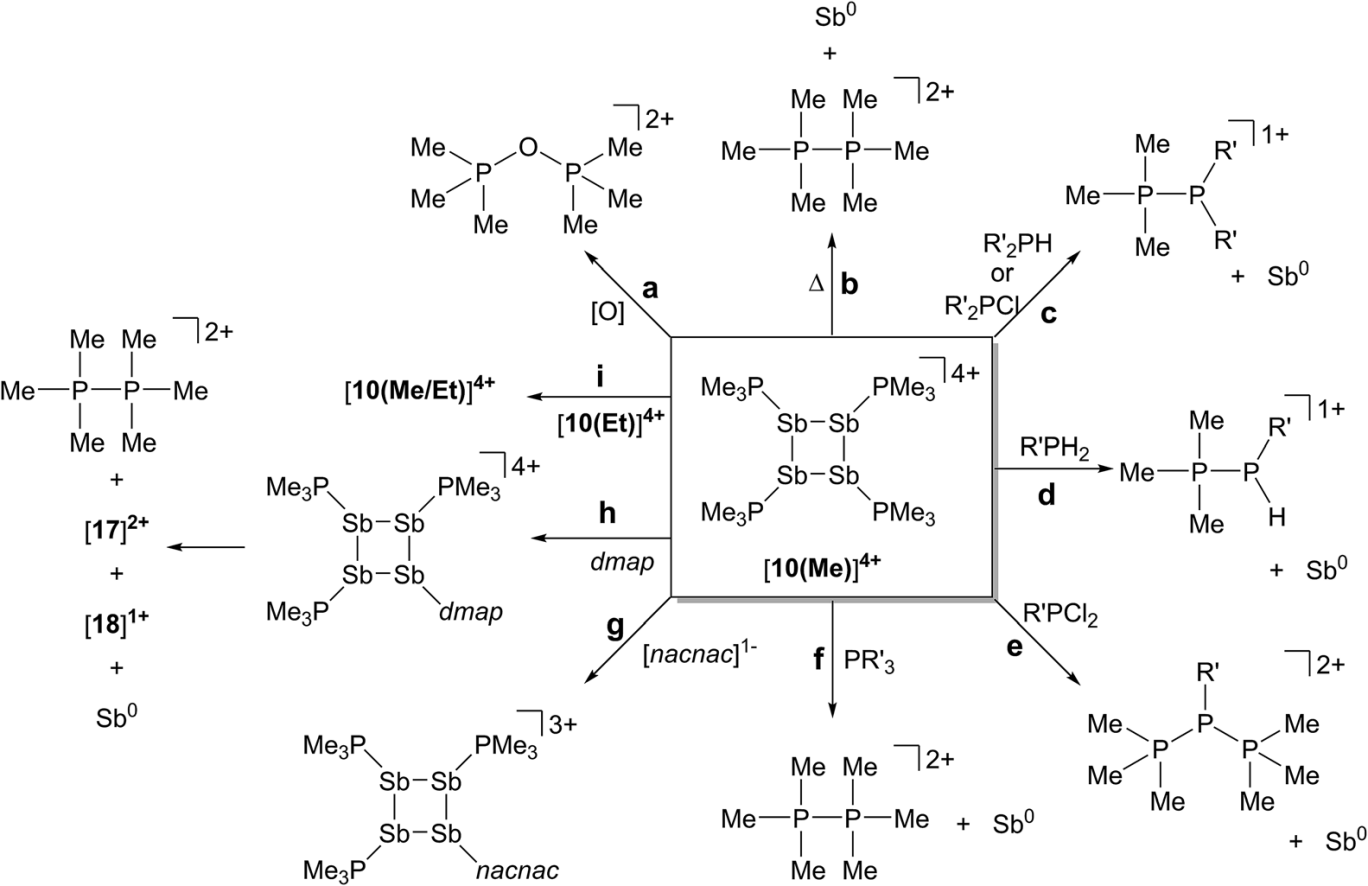
Reactivity of a prototypical *cyclo*-tetra(stibinophosphonium) tetracation, [**10(Me)**]^4+^. See text for descriptions of **a–i**.

The reductive elimination of diphosphonium dications [**11(R)**]^2+^ from trialkylphosphine complexes of highly electrophilic antimony(iii) centres is reported. The reduced antimony(i) fragments cyclize into frameworks identified as *cyclo*-tetra(stibinophosphonium) tetracations, [**10(R)**]^4+^. As outlined in Scheme 3, a phosphine catalyzed mechanism is proposed for fluoroantimony complexes, and isolation or spectroscopic characterization of key mechanistic intermediates is presented. The scope of this reductive assembly is dependent upon the steric bulk of the phosphine employed as demonstrated by non-productive reactions involving P^i^Pr_3_. Formation of cyclic (R-Pn)_*n*_ or [L-Pn]_*n*_^(*n*+)^ species (R = aryl group, L = alkylphosphine ligand, E = heavy pnictogen) appears to be the general fate of low-valent (R-Pn) or [L-Pn]^1+^ monomers, respectively. A multi-gram scale synthesis for the triflate salt of a prototypical *cyclo*-tetra(stibinophosphonium) tetracation, [**10(Me)**][OTf]_4_, has enabled reactivity studies that are summarized in Scheme 14.

In broad terms, the reactivity of *catena*-antimony(i) cation [**10(Me)**]^4+^ is directed by two features: (i) high charge concentration, and (ii) the presence of strongly polarized P–Sb bonds. The former explains the electrophilicity of cation [**10(Me)**]^4+^, its thermolysis to extrude [**11(Me)**]^2+^, and the observed facility for reductive elimination to yield elemental antimony (Scheme 14, reactions **a–f**). The significant polarization of the P–Sb bonds enables activation of a wide spectrum of bonds with the unusual outcome of yielding the same products *via* reaction with oppositely polarized substrates (*e.g.* P–Cl and P–H containing reagents) (Scheme 14, reactions **c–f**). This unique feature has led to the spectroscopic detection of the an H-phosphino-phosphonium cation, [Me_3_PP(H)Cy]^1+^, examples of which have not been reported previously. The high P–Sb bond polarization also supports a coordinate bonding model, consistent with ligand displacement reactivity demonstrated for cation [**10(Me)**]^4+^ (Scheme 14, reactions **g–i**). Ligand displacement has permitted functionalization of the four-membered Sb ring with substituents such as [*nacnac*]^1–^ or *dmap* (transiently). A heteroleptic phosphine substitution pattern around the Sb_4_ is feasible, but multiple isomers are observed on a relatively shallow potential energy surface hindering the isolation of a single derivative.

Within the broader context of phosphines as ubiquitous ligands in coordination chemistry, evidence of a novel ligand activation pathway has been presented and the associated reactants and products characterized. Taken together with previous, albeit less definitive, detection of such reactivity,^10,42^ the observation of this reductive elimination pathway confirms that these prototypical ligands can behave simultaneously as reducing agents and stabilizing ligands, a feature that may be generally applicable for phosphine complexes of highly electrophilic acceptors across the periodic table. Diversification of this synthetic protocol may therefore provide access to more extensively catenated systems for antimony as well as other elements. As demonstrated for [**10(Me)**]^4+^, a unique and rich reaction chemistry can be expected, in addition to the potential for valuable emergent properties such as σ-bond conjugation and cooperative catalysis due to metal catenation.
