# Supplementary material for: Synthesis and reactivity of cyclo-tetra(stibinophosphonium) tetracations: redox and coordination chemistry of phosphine–antimony complexes
Source: Chem Sci. 2015 Feb 3;6(4):2559–74. doi: 10.1039/c4sc03939d (PMC5647858; doi:10.1039/c4sc03939d)
Supplement: Supplementary file 1 [file SC-006-C4SC03939D-s001.pdf]

Electronic Supplementary Material (ESI) for Chemical Science.
This journal is © The Royal Society of Chemistry 2015

Synthesis and Reactivity of *Cyclo*-tetra(stibinophosphonium) Tetracations: Redox and Coordination Chemistry of Phosphine-Antimony Complexes

Saurabh S. Chitnis,[†] Alasdair P. M. Robertson,[†] Neil Burford,[*†] Jan J. Weigand,[*‡] and Roland Fischer[#]

[†] Department of Chemistry, University of Victoria, Victoria, BC V8W 3V6, Canada. Fax: +1 250 721 7147; Tel: +1 250 721 7150; E-mail: nburford@uvic.ca

[‡] Department of Chemistry and Food Chemistry, TU Dresden, 01062, Dresden, Germany. Tel: +49 351 46842800; E-mail: jan.weigand@tu-dresden.de

Institute for Inorganic Chemistry, TU Graz, 98010, Graz, Austria. Tel: +43 316 87332109; E-mail: roland.fischer@tugraz.net

SUPPLEMENTARY INFORMATION

Experimental Details 3

Figure S1. ^{31}P NMR spectrum of an equimolar mixture of [**11(Me)**][OTf]$_2$ and anhydrous CsF in CD_3CN. 13

Figure S2. $^{31}P\{^1H\}$ NMR spectrum of reaction mixture containing [$(Me_3P)_2SbCl$][OTf]$_2$ and 20 mol % PMe_3 in CD_3CN. 13

Figure S3. Molecular structure of [**13**][OTf]$_2$ in the solid state. Hydrogen atoms have been omitted for clarity. Thermal ellipsoids are drawn at 30 % probability level. Bond lengths (Å) and angles (°) are as follows: Sb-F = 1.952(1), Sb-P1 = 2.5575(6), Sb-P2 = 2.5846(6), Sb-O1 = 2.575(2), Sb-O3 = 2.956(3), Sb-Sb' = 5.1812(6), P1-Sb-P2 = 80.08(2), F-Sb-P1 = 85.92(5), F-Sb-P2 = 81.02(4), F-Sb-O1 = 153.58 (6). 14

Figure S4. $^{31}P\{^1H\}$ (left) and $^{19}F\{^1H\}$ (right) NMR resonances for [**13**][OTf]$_2$ in CD_3CN at 298 K. ...14

Figure S5. Solid-state structure of [**8(Me)**][**11(Me)**][OTf]$_5$. Hydrogen atoms and one MeCN molecule has been omitted for clarity. Thermal ellipsoids are drawn at 30 % probability level. 15

Figure S6. ^{31}P NMR chemical shifts of derivatives of [**7(R)**]$^{2+}$, [8(R)]$^{3+}$, [10(R)]$^{4+}$, [11(R)]$^{2+}$, [12(R)]$^{1+}$, and PR_3 (far right) relative to the PMe_3 containing derivative of each species (Δ, in ppm)...15

Figure S7. Raman spectrum (100 mW, 298 K) of black residue obtained upon thermolysis of [**10(Me)**][OTf]$_4$. 16

Figure S8. ^1H NMR spectrum (CD_3CN, 298 K, 500 MHz) of reaction mixture containing $CyPH_2$ and [**10(Me)**][OTf]$_4$ in a 2:1 ratio. 16

Figure S9. $^{31}P\{^1H\}$ NMR spectra (CH_3CN, 298 K) showing decomposition of a MeCN solution of [**15(Me)**][OTf]$_3$ at room temperature over 120 hours..17

Figure S10. Molecular structure of (*nacnac*)Sb(OTf)$_2$ in the solid state. Non-essential hydrogen atoms have been omitted for clarity. Thermal ellipsoids are drawn at 30 % probability level. Bond lengths (Å) and angles (°) are as follows: N1-Sb = 2.071 (2), N2-Sb = 2.075 (2), O1-Sb = 2.287 (1), O2-Sb = 2.196(1), N1-Sb-N2 = 89.11(6), O1-Sb-O2 = 174.33(5), O1-Sb-N1 = 88.86(6), O1-Sb-N2 = 89.46(6), O2-Sb-N1 = 86.31(6), O2-Sb-N2 = 87.51(6). 17

Figure S11. ^{31}P NMR spectrum (CH_3CN, 298 K, 202.5 MHz) of an equimolar reaction mixture containing *dmap* and [**11(Me)**]$^{2+}$ after 4 hours of stirring at room temperature.....................................18

Table S1. Crystallographic data for compounds [**13**][OTf]$_2$, [**11(Et)**][OTf]$_2$, [**12(Me)**][OTf], [**10(Et)**][OTf]$_4$•(MeCN), [**15(Me)**][OTf]$_3$•(MeCN), and [(*nacnac*)SbOTf$_2$]..18

Experimental Details

All manipulations were carried out using standard Schlenk or glovebox techniques under strict exclusion of oxygen and water in atmospheres of dried argon or nitrogen. Solvents were dried by distillation from CaH_2 (MeCN, CH_2Cl_2, fluorobenzene) or NaK alloy (THF, Et_2O, hexane, pentane). Deuterated solvents were obtained from Sigma Aldrich. All solvents were stored over molecular sieves (3 Å: MeCN, CD_3CN, 4 Å: fluorobenzene, CH_2Cl_2, CD_2Cl_2) or potassium mirror (THF, Et_2O, hexane, pentane). All glassware was dried in an oven at 160 °C or heat dried under vacuum. Melting points were recorded on an electrothermal melting point apparatus (Barnstead Electrothermal IA9100) in sealed capillaries under Argon atmosphere and are uncorrected. Infrared (IR) and Raman spectra were recorded at ambient temperature using a Bruker Vertex 70 instrument equipped with a RAM II module (Nd:YAG laser, 1064 nm). Selected IR spectra were recorded on a Perkin Elmer Frontier instrument. In all cases an ATR unit (diamond) was used for recording IR spectra. IR intensities are reported relative to the most intense peak and are given in parentheses using the following abbreviations: vw = very weak, w = weak, m = medium, s = strong, vs = very strong. Raman frequencies are accompanied by ranked intensities in parentheses. Nuclear magnetic resonance (NMR) spectra were obtained using various spectrometers and the field strengths as well as measurement temperature are explicitly given with the chemical shift values for each compound. Chemical shifts were referenced to 85% H_3PO_4 (^{31}P), $SiMe_4$ (^{1}H, ^{13}C), and $CFCl_3$ (^{19}F). Samples for NMR analysis were prepared in J-Young tubes or in standard tubes sealed with Teflon tape and Parafilm. SbF_3 (99.8 %) was obtained from Sigma Aldrich and sublimed prior to use. All liquid phosphines were distilled prior to use. Trimethylsilyl trifluoromethanesulfonate (TMSOTf) was obtained from Sigma Aldrich (99 %) and distilled prior to use. Li[*nacnac*$^{(dipp)}$] was prepared according to published procedures.[1] Elemental analyses were performed at MEDAC LTD in Chobahm, Surrey, UK or at Canadian Microanalytical Service in Delta, British Columbia, Canada.

FSb(OTf)$_2$: Into a rapidly stirred suspension of SbF_3 (41.4 mmol, 7.400 g) in MeCN (20 mL) was dropwise cannulated neat TMSOTf (83 mmol, 18.443 g) over 10 minutes, to obtain a clear, colourless solution. The reaction mixture was allowed to stir for 16 hours while attached to an oil bubbler to vent gaseous Me_3SiF evolved during the course of the reaction. All volatiles were removed under dynamic vacuum at room temperature yielding a white solid. After crushing this crude product into a fine powder, dynamic vacuum at 100 °C was applied until an IR assay showed no evidence of residual MeCN. The fine, colourless powder was used without further purification. Yield: 17.975 g; Melting

point: 260-263 °C; IR (ATR, 298 K, [cm^{-1}]): 219 (m), 227 (m), 251 (w), 297 (w), 331 (m), 344 (m), 365 (m), 511 (s), 582 (s), 587 (s), 623 (vs), 632 (m), 668 (vw), 775 (w), 964 (vs), 974 (vs), 1020 (s), 1034 (w), 1100 (s), 1127 (s), 1200 (vs), 1229, (m), 1311 (s), 1455 (vw); Raman (110 mW, RT, [cm^{-1}]): 228.8 (12), 255.5 (14), 324.1 (7), 347.8 (6), 368.9 (13), 515.2 (16), 582.0 (5), 600.2 (2), 649.9 (16), 775.6 (1), 938.5 (22), 983.5 (21), 1018.7 (9), 1033.6 (8), 1104.1 (15), 1131.6 (3), 1173.2 (20), 1207.7 (17), 1228.5 (10), 1313.8 (4), 1361.0 (18), 2266.7 (4), 2295.7 (19), 2944.7 (11), 3011.5 (23); $^{13}C\{^{1}H\}$ (CD_3CN, 298 K, 125.8 MHz, δ [ppm]): 120.6 (*quartet*, $^{1}J_{CF}$ = 318 Hz, CF_3); ^{19}F NMR (CD_3CN, 298 K, 282.4 MHz, δ [ppm]): -95.3 (broad *singlet*, SbF) -78.7 (*singlet*, CF_3).

$Sb(OTf)_3$: Into a rapidly stirred suspension of SbF_3 (50 mmol, 8.938 g) in MeCN (40 mL) was dropwise cannulated neat TMSOTf (150 mmol, 33.333 g) over five minutes yielding a clear effervescent solution. The reaction mixture was allowed to stir for 24 hours under argon while attached to an oil bubbler to vent gaseous Me_3SiF formed during the reaction. Removal of volatiles under dynamic vacuum at room temperature yielded a cream-coloured solid. The highly moisture and air-sensitive material was crushed into a fine powder and placed under dynamic vacuum at 80 °C until an IR assay showed no remaining MeCN. The product was obtained as a fine pale yellow powder and was used without further purification. Yield: 27.312 g; Melting point: 181 °C dec.; IR (ATR, 298 K, [cm^{-1}]): 240 (w), 255 (m), 326 (m), 344 (m), 359 (s), 399 (w), 511 (s), 573 (m), 588 (s), 618 (vs), 772 (m), 799 (s), 939 (vs), 958 (vs), 981 (vs), 1037 (vw), 1130 (vs), 1147 (s), 1195 (vs), 1323 (m), 1364 (w); Raman (101 mW, RT, [cm^{-1}]): 75.2 (2), 117.3 (4), 144.9 (15), 180.3 (14), 266.4 (3), 326.7 (7), 388.2 (12), 536.6, 574.4 (13), 602.8 (9), 647.0 (10), 778.2 (1), 894.4 (16), 1013.4 (9), 1108.6 (5), 1132.2 (8), 1217.9 (6), 1245.0 (9), 1323.2 (11), 1374.9 (17), 1408.2 (18); $^{13}C\{^{1}H\}$ (CD_3CN, 298 K, 125.8 MHz, δ [ppm]): 120.5 (*quartet*, $^{1}J_{CF}$ = 318 Hz, CF_3) ; ^{19}F NMR (CD_3CN, 298 K, 282.4 MHz, δ [ppm]): -78.5 (*singlet*, CF_3).

$[(PMe_3)_2SbF][OTf]_2$ [7(Me)][OTf]$_2$: To a clear solution of $FSb(OTf)_2$ (1 mmol, 0.439 g) in MeCN (5 mL) was dropwise added a solution of PMe_3 (2 mmol, 0.152 g) in MeCN (2 mL) yielding a white suspension. The suspension was allowed to stir for 15 minutes and then allowed to settle for 30 minutes. The clear, pale-yellow coloured supernatant was removed and the remaining white precipitate washed with MeCN (2 mL) and Et_2O (2 mL). Removal of volatiles under dynamic vacuum over 2 hours yielded [**7(Me)**][OTf]$_2$ as an analytically pure powder. Yield: 0.443 g (75 %); Melting Point: 170 °C dec.; Elemental Analysis (calcd./expt.): C (16.26/15.87) H (3.07/3.04); IR (ATR, 298 K, [cm^{-1}]):

229 (m), 269 (vw), 318 (w), 350 (m), 514 (vs), 546 (s), 572 (m), 633 (vs), 758 (w), 788 (vw), 851 (w), 953 (s), 1016 (vs), 1155 (vs), 1202 (s), 1223 (s), 1243 (s), 1257 (s), 1301 (m), 1419 (w), 2850 (vw), 2920 (vw), 3000 (vw); Raman (35 mW, RT, [cm^{-1}]): 199.0 (9), 223.9 (15), 269.7 (12), 319.1 (10), 348.4 (5), 516.6 (16), 546.7 (8), 574.7 (11), 638.4 (16), 671.1 (6), 761.9 (3), 955.4 (17), 974.9 (17), 1016.0 (16), 1030.6 (2), 1174.1 (13), 1223.7 (7), 1292.5 (16), 1313.7 (17), 1421.6 (8), 2801.8 (17), 2833.4 (17), 2880.7 (17), 2922.6 (1), 2995.6 (4), 3025.4 (14); ^{1}H NMR (CD_3CN, 298K, 500.3 MHz, δ [ppm]): 2.2 (broad *doublet*, $^{2}J_{HP}$ = 14.1 Hz, CH_3); ^{13}C{^{1}H} (CD_3CN, 298 K, 125.8 MHz, δ [ppm]): 9.7 (*doublet*, $^{1}J_{CP}$ = 32 Hz, CH_3), 121.3 (*quartet*, $^{1}J_{CF}$ = 321 Hz, CF_3); ^{31}P{^{1}H} (CD_3CN, 298 K, 202.5 MHz, δ [ppm]): 15.9 (*doublet*, $^{2}J_{PF}$ = 44 Hz); ^{19}F{^{1}H} NMR (CD_3CN, 298 K, 282.5 MHz, δ [ppm]): -176.3 (*triplet*, $^{2}J_{PF}$ = 44 Hz, SbF), -79.0 (*singlet*, CF_3).

[$(PEt_3)_2$SbF][OTf]$_2$, [7(Et)][OTf]$_2$: To a clear solution of FSb(OTf)$_2$ (1 mmol, 0.439 g) in MeCN (5 mL) was dropwise added a solution of PEt_3 (2 mmol, 0.236 g) in MeCN (2 mL) yielding a pale-yellow coloured, clear solution. The solution was allowed to stir for further 30 minutes. The clear reaction mixture was filtered, layered with Et_2O and placed in the freezer to yield very fine, colourless needles over the course of 72 hours. Removal of solvent under dynamic vacuum yielded [**7(Et)**][OTf]$_2$ as an analytically pure fine white powder. Yield: 0.478 g (71 %); Melting Point: 93-95 °C dec.; Elemental Analysis (calcd./expt.): C (24.90/24.65) H (4.48/4.46); IR (ATR, 298 K, [cm^{-1}]): 227 (m), 320 (w), 349 (m), 360 (m), 512 (vs), 573 (m), 741 (m), 775 (m), 632 (vs), 878 (w), 1010 (vs), 1045 (m), 1150 (s), 1171 (s), 1202 (s), 1228 (s), 1294 (m), 1395 (w), 1417 (w), 1464 (m), 2885 (vw), 2947 (w), 2981 (vw); Raman (35 mW, RT, [cm^{-1}]): 213.7 (9), 241.9 (10), 317.4 (10), 349.2 (10), 381.8 (14), 424.9 (15), 458.3 (18), 529.0 (11), 575.6 (13), 613.0 (7), 679.4 (23), 707.5 (22), 762.8 (6), 988.3 (17), 1020.4 (3), 1044.7 (8), 1167.3 (19), 1200.2 (19), 1229.0 (12), 1277.0 (20), 1298.0 (20), 1312.8 (20), 1395.3 (20), 1413.5 (16), 1464.4 (8), 2751.9 (21), 2765.7 (21), 2887.8 (5), 2920.9 (2), 2947.3 (1), 2985.7 (4); ^{1}H NMR (CD_3CN, 298 K, 500.3 MHz, δ [ppm]): 1.36 (*doublet of triplets*, CH_3,$^{3}J_{HH}$ = 7.8 Hz, $^{3}J_{HP}$ = 18.8 Hz, 18H), 2.51 (*doublet of quartets*, CH_2,$^{3}J_{HH}$ = 7.8 Hz, $^{2}J_{HP}$ = 8.7 Hz, 12H); ^{13}C{^{1}H} NMR (CD_3CN, 298 K, 125.8 MHz, δ [ppm]) : 8.2 (*doublet*, CH_3 ,$^{2}J_{CP}$ = 7 Hz), 15.4 (*doublet*, CH_2 ,$^{1}J_{CP}$ = 26 Hz), 121.3 (*quartet*, $^{1}J_{CF}$ = 321 Hz, CF_3); ^{31}P NMR (CD_3CN, 298 K, 202.5 MHz, δ [ppm]): 38.6 (*s*); ^{19}F{^{1}H} NMR (CD_3CN, 298 K, 282.5 Hz, δ [ppm]): -175.2 (broad *singlet*, SbF), -79.0 (*singlet*, CF_3).

*Note: The syntheses of salts [**10(R)**][OTf]$_4$ require rigorously dried and deoxygenated reagents, solvents, and inert gas atmospheres. Contamination of the system by oxygen leads to the formation of a black residue which, if allowed to mix with the product, is inseparable from the mixture.*

[(PMe$_3$)$_4$Sb$_4$][OTf]$_4$, [10(Me)][OTf]$_4$: Freshly sublimed SbF_3 (48 mmol, 8.531 g) was added to a 250 mL 3-necked schlenk flask equipped with a Teflon stir bar. The flask was attached to the Schlenk manifold and appended with an oil bubbler (to vent formed Me_3SiF) and a Schlenk frit (G3) with a 100 mL receiving flask. The apparatus was purged 3 times with fresh argon and then 55 mL of rigorously dried and deoxygenated MeCN was added via syringe to obtain a white suspension. Freshly distilled TMSOTf (96 mmol, 17.4 mL) was added via syringe over the course of 15 minutes and the resulting clear solution was allowed to stir for 24 h at room temperature to generate a clear solution of FSb(OTf)$_2$. The solution was concentrated to a volume of *ca.* 40 mL and cooled to 0 °C by applying an ice bath. Under rapid stirring, freshly distilled PMe_3 (106 mmol, 10.8 mL) was slowly injected under the surface of the solution (to minimize evaporative loss of PMe_3) with a syringe over a span of 5 minutes. A yellow-coloured suspension was obtained by the end of addition, which was allowed to stir for a further 0.5 h at 0 °C.* The reaction mixture was filtered via the attached Schlenk frit to yield a fine, yellow-coloured powder and a yellow-orange filtrate in the receiving flask. The frit was separated from the receiving flask and the yellow powder was dried under vacuum. The filtrate was concentrated to *ca.* 50 % of its original volume and cooled to 0 °C. Thus, a second crop of yellow-coloured powder, which was isolated by carefully removing the dark orange supernatant via a filter-tipped cannula, was obtained. The combined crops were dried under vacuum. A ^{31}P NMR assay showed the presence of a small amount of [**12(Me)**][OTf] which was removed washing with cold MeCN (15 mL). After drying for 16 h under dynamic vacuum at room temperature, [(PMe$_3$)$_4$Sb$_4$][OTf]$_4$ was obtained as a fine yellow powder. Yield: 13.87 g, (83 %). A complete characterization for this compound has been reported previously.[2]

*During stirring, occasionally, the suspension starts to turn dark orange. This is a sign that the concentration of free PMe_3 (reducing agent) in the mixture is too high, resulting in the formation of elemental antimony. It is vital to immediately apply slight vacuum to remove some of the volatile phosphine.

[(PEt$_3$)$_4$Sb$_4$][OTf]$_4$, [10(Et)][OTf]$_4$: A solution of FSb(OTf)$_2$ in 60 mL of MeCN was generated as described above using 8.8951 g (50 mmol) of SbF_3 and 18 mL (100 mmol) of TMSOTf. The solution

was concentrated to a volume of *ca.* 45 mL and cooled to 0 °C by applying an ice bath. With rapid stirring, PEt_3 (105 mmol, 12.3 mL) was injected over 2 minutes. The resulting slightly yellow-coloured reaction mixture was stirred for an additional 15 minutes at 0 °C and then warmed to room temperature. Stirring was continued for 24 hours and the resulting clear, orange-coloured solution was placed under vacuum with stirring until the formation of large amount of yellow-coloured precipitate was observed. This yellow precipitate was separated by filtration (G3 frit) from the orange supernatant. The frit was removed from the receiving flask and the residue dried under vacuum. A second crop of product was optained by concentrating the dark orange-coloured filtrate and separating the mother liquor via a filter-tipped cannula to give a yellow-coloured precipitate. The combined crops were dried under vacuum to yield a very bright yellow-coloured powder (12.105 g). A ^{31}P NMR assay of the product indicated trace amounts of [**12(Et)**][OTf]. The product was recrystallized from a minimum amount of thoroughly dried MeCN and washed with fluorobenzene (2 x 25 mL) and Et_2O (2 x 25 mL). Application of dynamic vacuum over the course of 16 hours at room temperature yielded analytical grade [**10(Et)**][OTf]$_4$ as a bright yellow-coloured powder. Yield: 9.505 g, (49 %); Melting Point: 161.2 °C dec.; Elemental Analysis (calcd./expt.): C (21.62/21.76) H (3.89/4.26); Raman (50 mW, RT, [cm^{-1}]): 186.4 (5), 218.1 (7), 267.1 (16), 315.4 (10), 349.3 (9), 374.8 (15), 427.2 (17), 457.8 (18), 517.8 (22), 574.7 (12), 620.5 (13), 684.7 (24), 720.4 (24), 759.7 (8), 978.0 (14), 1030.0 (3), 1169.5 (23), 1185.0 (24), 1227.0 (11), 1289.9 (18), 1385.6 (20), 1412.7 (15), 1466.1 (10), 2252.2 (25), 2745.6 (19), 2760.3 (20), 2812.2 (21), 2880.8 (6), 2911.4 (1), 2944.6 (2), 2979.5 (4); ^{1}H NMR (CD_3CN, 300K, 400.1 MHz, δ [ppm]): 1.34 (*multiplet*, 12H), 2.6 (*multiplet*, 8H); $^{13}C\{^{1}H\}$ NMR (CD_3CN, 300 K, 100.6 MHz, δ [ppm]): 8.2 (*singlet*, CH_3), 16.8 (*multiplet*, CH_2), 120.8 (*quartet*, $^{1}J_{CF}$ = 319 Hz, CF_3); ^{31}P NMR (CD3CN, 300 K, 161.9 Hz): 8.8 (*singlet*); ^{19}F NMR (CD_3CN, 300 K, 376.5 MHz, δ [ppm]): -79.1 (*singlet*, CF_3).

[Et$_3$PPEt$_3$][OTf]$_2$, [11(Et)][OTf]$_2$: Ph_3SbCl_2 (0.43 mmol, 0.183 g) and AgOTf (0.86 mmol, 0.222 g) were combined under the exclusion of light and 5 mL CH_2Cl_2 was added yielding which resulted in the formation of a white suspension. The reaction mixture was stirred for additional 60 minutes under the exclusion of light and then filtered to remove the formed AgCl. To the clear and colourless filtrate neat PEt_3 (0.86 mmol, 0.101 g) was dropwise added, which resulted in the formation of a white suspension. After stirring for 15 minutes the colourless precipitate was isolated by filtration and washed with CH_2Cl_2 (2 mL). After removing of all volatiles under dynamic vacuum [**11(Et)**][OTf]$_2$ was obtained as an analytically pure colourless powder. Yield: 0.212 g (46 %); Melting Point: 169-170 °C; Elemental

Analysis (calcd./expt.): C (31.46/31.68) H (5.66/5.75); IR (ATR, 298 K, [cm^{-1}]): 210 (w), 228 (w), 248 (w), 347 (m), 421 (w), 516 (vs), 572 (s), 633 (vs), 698 (w), 734 (w), 755 (m), 780 (m), 877 (w), 961 (m), 1025 (vs), 1050 (w), 1149 (vs), 1217 (s), 1256 (s), 1306 (w), 1394 (w), 1465 (w), 2889 (vw), 2917 (w), 2954 (w), 2990 (vw); Raman (35 mW, RT, [cm^{-1}]): 262.0 (9), 313.2 (7), 348.1 (4), 387.3 (20), 422.3 (21), 504.6 (15), 519.6 (18), 574.8 (10), 595.1 (21), 625.5 (8), 683.9 (23), 726.4 (22), 757.2 (5), 789.4 (24), 973.8 (17), 994.9 (16), 1030.3 (1), 1056.5 (14), 1162.8 (21), 1223.1 (12), 1273.0 (18), 1384.2 (18), 1400.1 (15), 1454.2 (13), 1469.3 (11), 2760.9 (19), 2889.1 (6), 2907.7 (3), 2920.9 (3) , 2957.6 (2), 2994.5 (6), 3004.0 (8); ^{1}H NMR (CD_3CN, 298 K, 500.3 MHz, δ [ppm]): 1.43 (*m*, 18H), 2.84 (*m*, 12H); ^{13}C{^{1}H} NMR (CD_3CN, 298 K, 125.8 MHz, δ [ppm]): 8.1 (*singlet*, CH_3), 12.2 (*triplet*, $^{1}J_{CP}$ = 17 Hz, CH_2), 125.1 (*quartet*, $^{1}J_{CF}$ = 321 Hz, CF_3); ^{31}P{^{1}H} NMR (CD_3CN, 298 K, 202.5 MHz, δ [ppm]): 39.3 (*singlet*), ^{19}F{^{1}H} NMR (CD_3CN, 298 K, 282.5 MHz, δ [ppm]): -79.2 (*singlet*, CF_3). Crystals suitable for XRD were obtained as colourless blocks from a concentrated MeCN solution at -30 °C and exhibited the same ^{31}P NMR chemical shift as the bulk material.

Spectroscopic data for [Pr_3PPPr_3][OTf]$_2$, [11(Pr)][OTf]$_2$: The formation of this compounds in the reaction mixture was confirmed by an NMR assay of the reaction mixture containing PPr_3 and $Sb(OTf)_3$ in a 3:1 stoichiometry. The observed chemical shift shows an excellent match with reported NMR data.[3] ^{1}H NMR (CD_2Cl_2, 298 K, 300 MHz, δ [ppm]): 2.72-2.60 (*multiplet*, 12H), 1.82-1.65 (*multiplet*, 12H), 1.11 (*triplet of triplets*, $^{3}J_{HH}$ = 7 Hz, $^{4}J_{PH}$ = 1 Hz, 18H), ^{31}P NMR (CD_2Cl_2, 298 K, 146 MHz, δ [ppm]): 31.2 (*singlet*).

[(*dmpe*)SbF][OTf]$_2$, [13][OTf]$_2$: To a solution of FSb(OTf)$_2$ (3 mmol, 1.317 g) in MeCN (6 mL) was added a solution of *dmpe* (3 mmol, 0.450 g) in MeCN (2 mL) yielding a clear, pale yellow-coloured solution. The reaction mixture was allowed to stir for an additional 60 minutes after which a ^{31}P NMR assay showed complete consumption of *dmpe* (δ = -48.4 ppm) and the appearance of a broad doublet as the major phosphorus containing product. The reaction mixture was concentrated to 3 mL, filtered and placed in the freezer at -30 °C. A fine white powder precipitated, which was isolated by decanting the mother liquor and identified as analytically pure [**13**][OTf]$_2$. Yield: 1.152 g (65 %); Melting point: 104 °C dec.; Elemental Analysis (calcd./expt.): C (16.31/16.13) H (2.74/2.71); IR (ATR, 298 K, [cm^{-1}]): 218 (m), 226 (m), 246 (w), 268 (vw), 276 (vw), 303 (vw), 321 (w), 350 (m), 440 (w), 514 (vs), 541 (s), 575 (m), 631 (vs), 728 (w), 761 (w), 773 (w), 796 (vw), 819 (vw), 852 (m), 876 (m), 911 (m), 927 (m), 942 (s), 960 (m), 1012 (vs), 1021 (vs), 1114 (w), 1154 (vs), 1184 (s), 1207 (vs), 1232 (vs), 1280 (s),

1293 (s), 1304 (m), 1415 (w), 1430 (vw), 2923 (vw), 2959 (vw), 3000 (vw); Raman (35 mW, RT, [cm^{-1}]): 187.0 (4), 209.0 (8), 246.6 (11), 266.3 (14), 318.3 (5), 350.0 (3), 441.8 (19), 517.6 (17), 543.6 (11), 576.2 (12), 652.8 (10), 733.8 (13), 764.4 (6), 795.6 (19), 927.5 (18), 1025.2 (2), 1114.8 (19), 1167.3 (16), 1180.1 (15), 1196.0 (17), 1231.1 (11), 1305.1 (15), 1412.6 (10), 2816.4 (18), 2925.1 (1), 2957.9 (9), 2965.6 (9), 3000.0 (7), 3029.7 (15); ^{1}H NMR (CD_3CN, 298 K, 500.3 MHz, δ [ppm]): 1.86 (broad *doublet*, $^{2}J_{HP}$ = 12.4 Hz, 6H), 2.1 (broad *doublet*, $^{2}J_{HP}$ = 12.9 Hz, 6H), 2.75 (broad *multiplet*, 4H); ^{13}C{^{1}H} NMR (CD_3CN, 298 K, 125.8 MHz, δ [ppm]): 6.1 (*multiplet*), 7.2 (*multiplet*), 24.3 (*multiplet*), 120.1 (*quartet*, $^{1}J_{CF}$ = 320 Hz, CF_3); ^{31}P NMR (CD_3CN, 298 K, 202.5 MHz, δ [ppm]): 41.2 (broad *doublet*, $^{2}J_{PF}$ = 39 Hz); ^{19}F{^{1}H} NMR (CD_3CN, 298 K, 282.5 MHz, δ [ppm]): -186.3 (broad *triplet*, $^{2}J_{PF}$ = 41 Hz, SbF), -78.1 (*singlet*, CF_3). Fine, needle-like crystals suitable for XRD were obtained by diffusion of Et_2O vapour into a concentrated MeCN solution at -30 °C and exhibited the same ^{31}P NMR chemical shift as the bulk material.

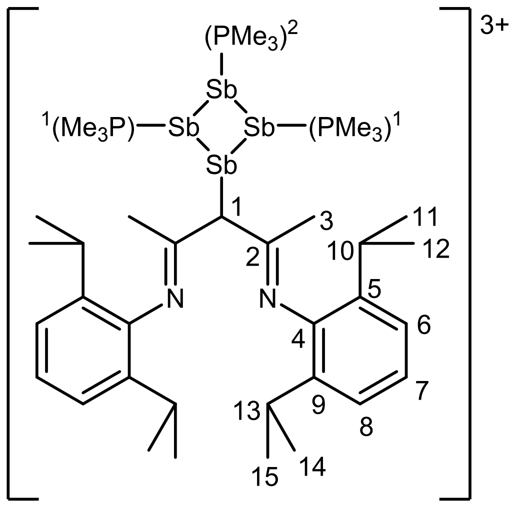

[(PMe_3)$_3$(*nacnac*^{dipp})Sb_4][OTf]$_3$, [15(Me)][OTf]$_3$: Solid [**10(Me)**][OTf]$_4$ (0.50 mmol, 0.692 g) and Li*nacnac*$^{(dipp)}$ (1.10 mmol, 0.467 g) were combined in a Schlenk flask equipped with a Teflon stir bar. The flask was attached to a Schlenk line and freshly distilled MeCN (10 mL) was added at 0 °C under argon with rapid stirring to yield an orange-coloured suspension. The flask was placed under a mild dynamic vacuum to effect removal of the evolved PMe_3. After stirring for four hours, the orange suspension was filtered to obtain a red-coloured filtrate, which was stored at -30 °C for 48 hours to yield a light brown-coloured precipitate. The precipitate was removed by filtration and washed with Et_2O (3 x 5 mL). After removal of all volatiles under dynamic vacuum, analytically pure **[15(Me)][OTf]**$_3$ was obtained as brown-coloured, powder. Yield: 0.412 g (52 %); Melting point: 164-165 °C, dec.; Elemental Analysis (calcd./expt.): The reactivity of the compound precluded the determination of satisfactory elemental analysis; Raman (30 mW, RT, [cm^{-1}]): 74.9 (2), 94.8 (3), 144.6 (1), 199.8 (5), 268.2 (17), 293.9 (22), 314.6 (20), 345.9 (12), 444.7 (28), 489.6 (27), 573.7 (26), 649.0 (13), 668.4 (14), 757.8 (9), 884.9 (23), 910.6 (29), 1025.9 (6), 1108.1 (18), 1163.5 (21), 1183.4 (10), 1213.1 (11), 1242.3 (16), 1312.1 (24), 1440.0 (19), 1464.0 (25), 1591.0 (7), 1645.4 (8), 2869.9 (30), 2912.9 (4), 3059.9 (15); ^{1}H NMR (CD_3CN, 298K, 400.1 MHz, δ [ppm]): 1.08 (*doublet*, $H^{11/12/14/15}$, $^{3}J_{HH}$

= 4.6 Hz, 6H), 1.10 (*doublet*, $H^{11/12/14/15}$, $^3J_{HH}$ = 4.6 Hz, 6H), 1.26 (*doublet*, $H^{11/12/14/15}$, $^3J_{HH}$ = 6.82 Hz, 6H), 1.28 (*doublet*, $H^{11/12/14/15}$, $^3J_{HH}$ = 6.75 H, 6H), 1.99 (*doublet*, $(PMe_3)^1$, $^2J_{HP}$ = 13.5 Hz, 18H), 2.07 (*s*, H^3, 6H), 2.27 (*doublet*, $(PMe_3)^2$, $^2J_{HP}$ = 14.39 Hz, 9H), 2.93 (*septet*, H^{10}, $^3J_{HH}$ = 6.75 Hz, 4H), 6.20 (*singlet*, H^1, 1H), 7.14-7.27 (broad *multiplet*, $H^{6/7/8}$, 6H); $^{13}C\{^1H\}$ NMR (CD_3CN, 298 K, 100.6 MHz, δ [ppm]): 14.0 (*doublet*, 2 x $(PMe_3)^1$, $^1J_{CP}$ = 34 Hz), 14.1 (*doublet*, $(PMe_3)^2$, $^1J_{CP}$ = 35 Hz), 23.9 (C^3), 24.0 ($C^{11/12/14/15}$), 24.1 ($C^{11/12/14/15}$), 24.4 ($C^{11/12/14/15}$), 25.2 ($C^{11/12/14/15}$), 29.0 ($C^{10/13}$), 29.1 ($C^{10/13}$), 57.0 (C^1), 124.6 ($C^{6/8}$), 121.9 (*quartet*, $^1J_{CF}$ = 320 Hz, CF_3) 124.9 ($C^{6/8}$), 126.0 (C^7), 137.6 ($C^{5/9}$), 138.4 ($C^{5/9}$), 174.6 (C^2); $^{31}P\ \{^1H\}$ NMR (CD_3CN, 300 K, 161.9 MHz, δ [ppm]): -33.6 (*doublet*, $(PMe_3)^1$, $^3J_{PP}$ = 32 Hz, 2P), -26.6 (*triplet*, $(PMe_3)^2$, $^3J_{PP}$ = 32 Hz, 1P); $^{19}F\{^1H\}$ NMR (CD_3CN, 298 K, 376.5 MHz, δ [ppm]): -79.1 (*singlet*, CF_3). Colourless block shaped crystals suitable for XRD were obtained upon recrystallization from a concentrated MeCN solution at -30 °C and exhibited the same ^{31}P NMR chemical shifts as the bulk material.

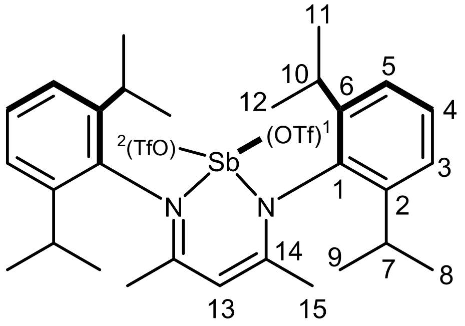

[(*nacnac*^{dipp})Sb(OTf)$_2$]: Solid $Sb(OTf)_3$ (11 mmol, 6.259 g) and Li*nacnac*$^{(dipp)}$ (11 mmol, 4.671 g) were combined in a Schlenk flask equipped with a Teflon stir bar. The flask was attached to a Schlenk line and 35 mL fluorobenzene was added under argon yielding an orange-coloured suspension. Upon stirring for 24 hours a dark orange-coloured suspension was obtained which was filtered *via* a glass frit (G3) to remove LiOTf. The resulting filtrate was concentrated to 10 mL, cooled to 0 °C and Et_2O (10 mL) was added under argon to yield a yellow suspension upon stirring for 20 minutes. This suspension was filtered *via* a glass frit (G3) and the residue washed with pentane (10 mL). Removal of all volatiles under dynamic vacuum at room temperature yielded the product as an analytically pure yellow-coloured powder. Yield: 6.171 g (67 %); Melting point: 144-145 °C, dec.; Elemental Analysis (calcd./expt.): C (44.46/43.62) H (4.93/4.72) N (3.34/3.61); Raman (60 mW, RT, [cm^{-1}]): 441.3 (7), 453.1 (6), 501.3 (15), 527.8 (9), 550.1 (2), 590.4 (14), 630.9 (17), 645.7 (14), 690.2 (19), 727.1 (21), 764.5 (5), 793.6 (23), 817.4 (21), 868.9 (24), 888.2 (10), 937.4 (26), 956.9 (22), 997.3 (6), 1031.9 (12), 1046.8, 1110.0 (12), 1165.9 (13), 1186.0 (4), 1244.2 (1), 1298.2 (11), 1320.5 (12), 1346.9 (17), 1371.2 (16), 1389.2 (17), 1442.7 (7), 1470.0 (9), 1530.6 (18), 1560.7 (20), 1590.7 (3), 2720.2 (28), 2766.3 (29), 2869.9 (25), 2913.6 (8), 2942.3 (7), 2968.9 (18), 2983.7 (26), 3031.6 (28), 3070.8 (27), 3170.8 (30); ^1H NMR (CD_2Cl_2, 300 K, 400.1 MHz, δ [ppm]): 1.21 (*doublet*, $H^{8/9/11/12}$, $^3J_{HH}$ = 6.8 Hz, 6H), 1.28 (*doublet*, $H^{8/9/11/12}$, $^3J_{HH}$ = 6.5 Hz, 6H), 1.35

(*doublet*, $H^{8/9/11/12}$, $^3J_{HH}$ = 6.9 Hz, 6H), 1.37 (*doublet*, $H^{8/9/11/12}$, $^3J_{HH}$ = 6.5 Hz, 6H), 2.16 (*singlet*, H^{15}, 6H), 2.98 (*septet*, $H^{7/10}$, $^3J_{HH}$ = 6.8 Hz, 2H), 3.36 (*septet*, $H^{7/10}$, $^3J_{HH}$ = 6.6 Hz, 2H), 5.91 (*singlet*, H^{13}, 1H), 7.36-7.38 (*multiplet*, $H^{3/5}$, 4H), 7.46-7.50 (*triplet*, H^4, $^3J_{HH}$ = 7.8 Hz, 2H); $^{13}C\{^1H\}$ NMR (CD_2Cl_2, 100.6 MHz, 300 K, δ [ppm]): 24.4 (C^{15}), 24.9 ($C^{8/9/11/12}$), 25.5 ($C^{8/9/11/12}$), 25.7 ($C^{8/9/11/12}$), 25.4 ($C^{8/9/11/12}$), 29.0 ($C^{7/10}$), 29.3 ($C^{7/10}$), 108.5 (C^{13}), 119.0 (*quartet*, $C^{OTf1/OTf2}$, $^1J_{CF}$ = 319 Hz), 120.3 (*quartet*, $C^{OTf1/OTf2}$, $^1J_{CF}$ = 317 Hz), 126.3 ($C^{3/5}$), 126.7 ($C^{3/5}$), 130.9 (C^4), 134.5 (C^1), 145.2 ($C^{2/6}$), 146.5 ($C^{2/6}$), 170.5 (C^{14}); $^{19}F\{^1H\}$ NMR (CD_3CN, 300 K, 376.5 MHz, δ [ppm]): -78.4 (*singlet*), -78.3 (*singlet*). Crystals suitable for XRD were grown from a saturated hexane solution at -30 °C and exhibited the same ^1H NMR chemical shifts as the bulk material.

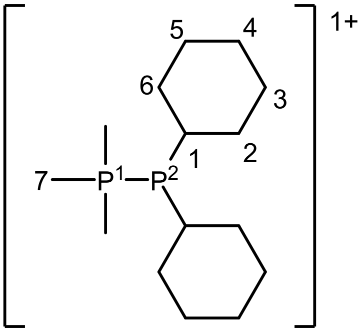

[Me_3PPCy_2][OTf]: To a bright yellow solution of [**10(Me)**][OTf]$_4$ (0.25 mmol, 0.347g) in MeCN (5 mL) was dropwise added neat Cy_2PCl (1 mmol, 0.233 g) yielding a black suspension. The reaction mixtures was stirred for a further 10 minutes and then allowed to settle yielding a clear and colourless supernatant above a fine black precipitate. A ^{31}P NMR assay of the supernatant showed quantitative consumption of [**10(Me)**][OTf]$_4$ and two coupled doublets indicating the formation of only one phosphorus containing species. The reaction mixture was filtered and all volatiles were removed under dynamic vacuum to yield a fine colourless powder. This powder was washed with Et_2O (2 x 1 mL) and dried to obtain analytically pure [Me_3PPCy_2][OTf]. Yield: 0.339 g (80 %); Melting point: 110-112 °C; Elemental Analysis (calcd./expt.): C (45.39/45.48) H (7.62/8.14); IR (ATR, 298 K, [cm^{-1}]): 297 (w), 335 (vw), 516 (s), 572 (w), 635 (vs), 678 (vw), 756 (vw), 849 (w), 889 (w), 958 (s), 1029 (s), 1151 (s), 1224 (s), 1255 (vs), 1282 (m), 1306 (w), 1323 (w), 2918 (m), 1420 (vw), 1449 (w), 2845 (w), 3000 (vw); Raman (35 mW, RT, [cm^{-1}]): 205.4 (13), 225.9 (6), 275.6 (11), 301.5 (13), 316.7 (11), 349.7 (11), 383.1 (13), 418.4 (10), 481.5 (10), 513.5 (13), 573.3 (12), 679.5 (6), 702.2 (9), 757.9 (10), 770.7 (13), 815.9 (8), 847.3 (10), 1002.1 (12), 1029.3 (3), 1081.1 (12), 1117.9 (13), 1173.5 (12), 1192.0 (13), 1226.0 (12), 1269.5 (10), 1297.8 (9), 1327.3 (12), 1344.5 (12), 1405.3 (11), 1421.8 (13), 1444.9 (4), 2710.8 (13), 2799.1 (13), 2848.3 (2), 2882.8 (5), 2919.7 (1), 2949.5 (6), 2974.1 (8), 3001.2 (7); ^1H NMR (CD_3CN, 298K, 500.3 MHz, δ [ppm]): 1.22-1.54 (broad *m*, 10H), 1.66-1.74 (*broad multiplet*, 2H), 1.75-1.86 (*broad overlapping multiplet*, $H^{2/6}$, 6H), 1.92 (*overlapping doublet of doublets*, 9 x H^7, $^2J_{PH}$ = 13.3 Hz, $^3J_{PH}$ = 2.7 Hz, 11H), 2.22 (*broad multiplet*, $H^{1/2}$, 2H); $^{13}C\{^1H\}$ NMR (CD_3CN, 298 K, 125.8 MHz, δ [ppm]): 12.3 (*doublet of doublets*, C^7, $^1J_{CP}$ = 43 Hz, $^2J_{CP}$ = 7 Hz), 26.6 (*singlet*, C^4), 27.9

(*doublet*, $C^{3/5}$, $^{3}J_{CP}$ = 10 Hz), 28.1 (*doublet*, $C^{3/5}$, $^{3}J_{CP}$ = 10 Hz), 32.1 (*doublet of doublets*, C^{1}, $^{1}J_{CP}$ = 11 Hz, $^{2}J_{CP}$ = 8 Hz), 32.9-33.4 (*overlapping multiplets*, $C^{2/6}$); ^{31}P NMR (CD_3CN, 298 K, 202.5 MHz, δ [ppm]): -5.7 (broad *doublet*, $^{1}J_{PP}$ = 328 Hz), 11.9 (*doublet of decets*, $^{1}J_{PP}$ = 328 Hz, $^{2}J_{HP}$ = 13 Hz); $^{19}F\{^{1}H\}$ NMR (CD_3CN, 298 K, 282.5 MHz, δ [ppm]): -79.3 (*singlet*, CF_3).

[Me_3PF][OTf], [12(Me)][OTf]: To a solution of PMe_3 (1 mmol, 0.076 g) inCH_2Cl_2 (5 mL) was added solid XeF_2 (1 mmol, 0.169 g) in 10 equal portions over 10 minutes. Effervescence was observed with each addition and a clear colourless solution was obtained by the end of the addition. The reaction mixture was allowed to stir for 20 minutes and then neat TMSOTf was added to obtain a white suspension. The suspension was allowed to stir for 15 minutes and then hexane was added to aid settling of the precipitate. After settling for 20 minutes at -30 °C, the clear and colourless supernatant was removed by pipette and the precipitate was dried under vacuum to yield analytically pure [**12(Me)**][OTf] as a very fine white powder. Yield: 0.201 g (82 %); Melting Point: 180-182 °C; Elemental Analysis (calcd./expt.): C (19.68/19.36) H (3.72/3.78); IR (ATR, 298 K, [cm^{-1}]): 218 (w), 254 (w), 297 (m), 318 (vw), 336 (m), 516 (s), 573 (m), 634 (vs), 668 (vw), 759 (w), 875 (s), 967 (vs), 994 (s), 1029 (s), 1153 (s), 1225 (vs), 1245 (s), 1306 (w), 1427 (w), 2918 (vw), 3000 (w), 3146 (vw); Raman (35 mW, RT, [cm^{-1}]): 260.0 (9), 288.2 (11), 316.7 (6), 336.4 (8), 351.6 (5), 521.0 (12), 574.7 (8), 640.2 (4), 674.8 (3), 760.0 (5), 783.6 (11), 878.6 (11), 972.7 (12), 1032.9 (2), 1228.6 (8), 1260.5 (10), 1410.1 (7), 1422.7 (7), 1885.1, 2923.8 (1), 3003.8 (4); ^{1}H NMR (CD_3CN, 298K, 300.3 MHz, δ [ppm]): 2.15 (*doublet of doublets*, $^{1}J_{HP}$ = 14.5 Hz, $^{2}J_{HF}$ = 12.6 Hz, CH_3); $^{13}C\{^{1}H\}$ NMR (CD_3CN, 298 K, 75.5 MHz, δ [ppm]) : 11.9 (*doublet of doublets*, $^{1}J_{CP}$ = 62 Hz, $^{2}J_{CF}$ = 9 Hz), 120.5 (*quartet*, $^{1}J_{CF}$ = 322 Hz, CF_3); $^{31}P\{^{1}H\}$ (CD_3CN, 298 K, 146.2 MHz, δ [ppm]): 145 (*doublet*, $^{1}J_{PF}$ = 951 Hz), $^{19}F\{^{1}H\}$ NMR (CD_3CN, 298 K, 282.5 MHz, δ [ppm]): -137.9 (*doublet*, $^{1}J_{PF}$ = 951 Hz, PF), -79.4 (*singlet*, CF_3). Colourless crystals suitable for XRD were obtained from a concentrated MeCN solution at -30 °C and exhibited the same ^{31}P NMR chemical shift as the bulk material.

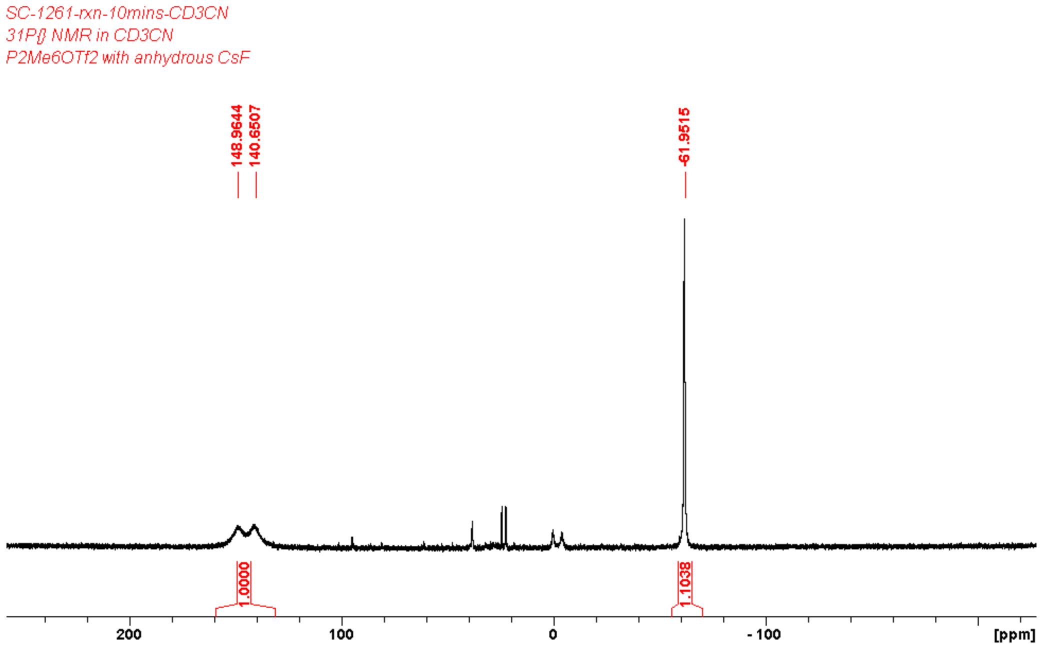

Figure S1. ^{31}P NMR spectrum of an equimolar mixture of [**11(Me)**][OTf]$_2$ and anhydrous CsF in CD_3CN.

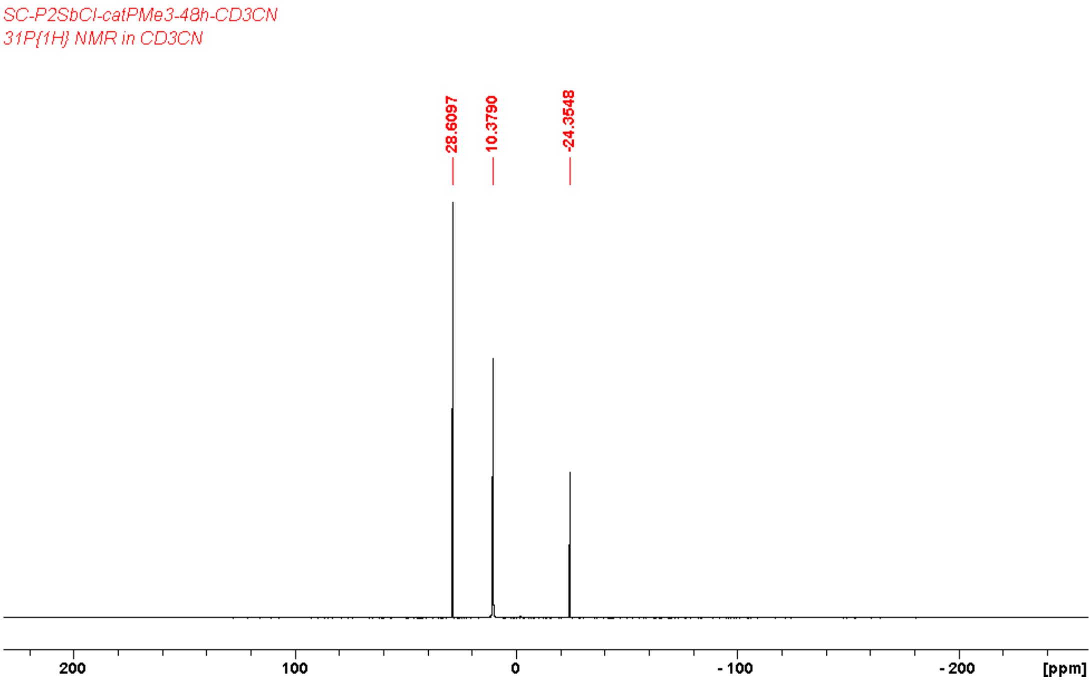

Figure S2. $^{31}P\{^1H\}$ NMR spectrum of reaction mixture containing $[(Me_3P)_2SbCl][OTf]_2$ and 20 mol % PMe_3 in CD_3CN.

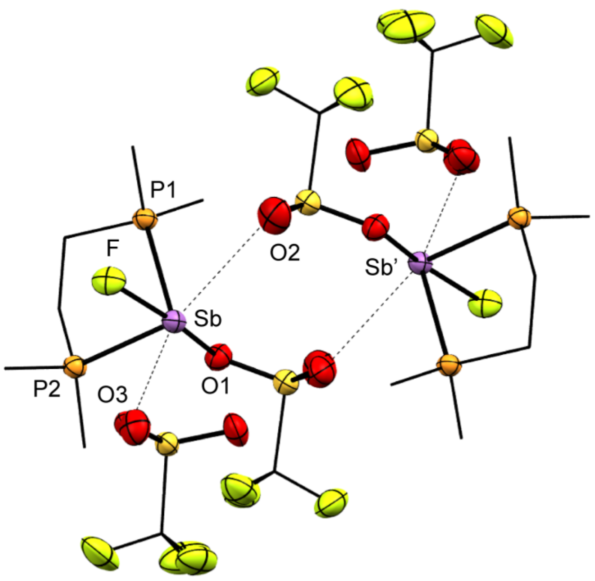

Figure S3. Molecular structure of [**13**][OTf]$_2$ in the solid state. Hydrogen atoms have been omitted for clarity. Thermal ellipsoids are drawn at 30 % probability level. Bond lengths (Å) and angles (°) are as follows: Sb-F = 1.952(1), Sb-P1 = 2.5575(6), Sb-P2 = 2.5846(6), Sb-O1 = 2.575(2), Sb-O3 = 2.003956(3), Sb-Sb' = 5.1812(6), P1-Sb-P2 = 80.08(2), F-Sb-P1 = 85.92(5), F-Sb-P2 = 81.02(4), F-Sb-O1 = 153.58 (6).

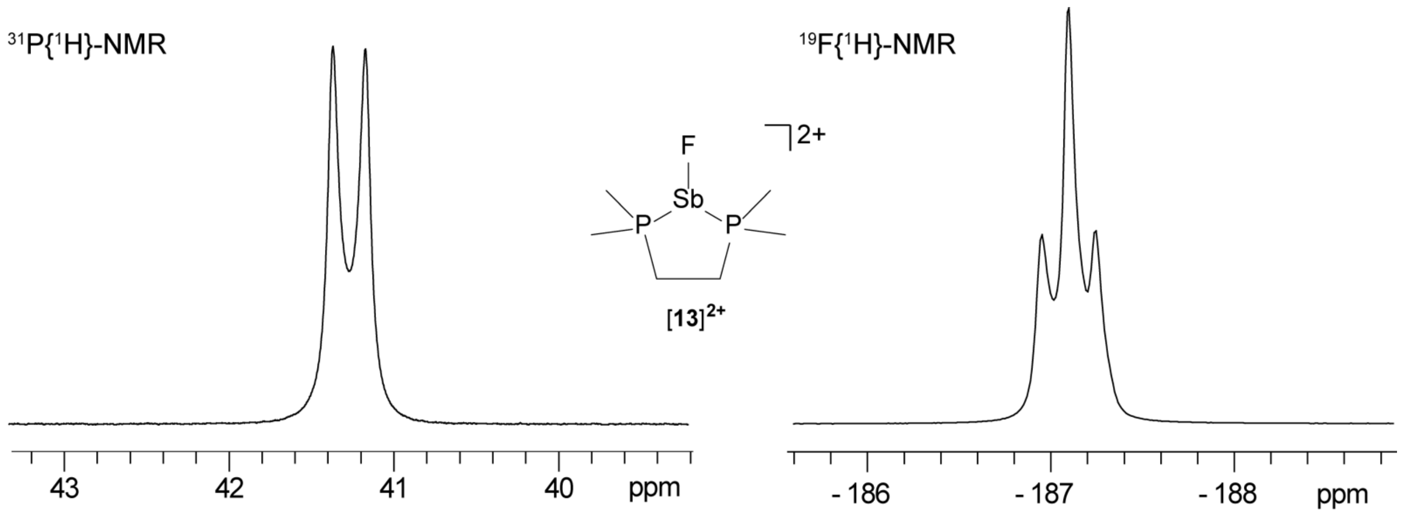

Figure S4. $^{31}P\{^1H\}$ (left) and $^{19}F\{^1H\}$ (right) NMR resonances for [**13**][OTf]$_2$ in CD_3CN at 298 K.

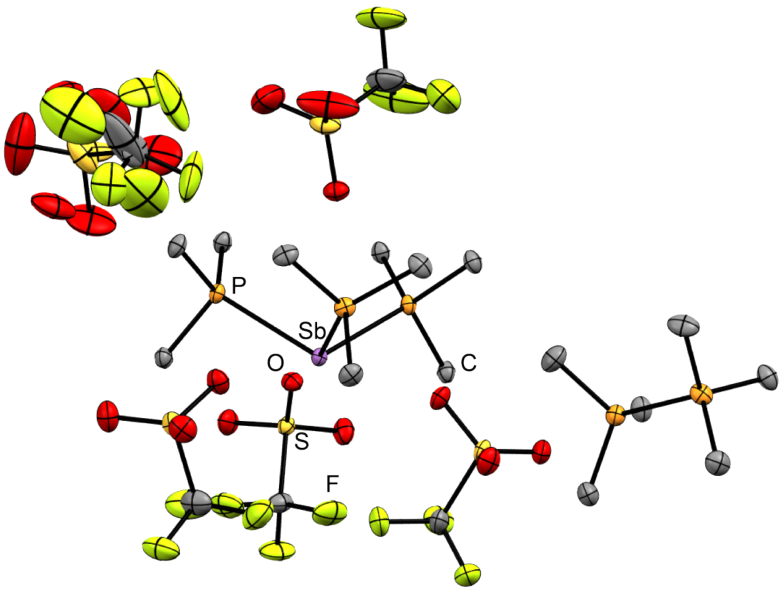

Figure S5. Solid-state structure of [**8(Me)**][**11(Me)**][OTf]$_5$. Hydrogen atoms and one MeCN molecule has been omitted for clarity. Thermal ellipsoids are drawn at 30 % probability level.

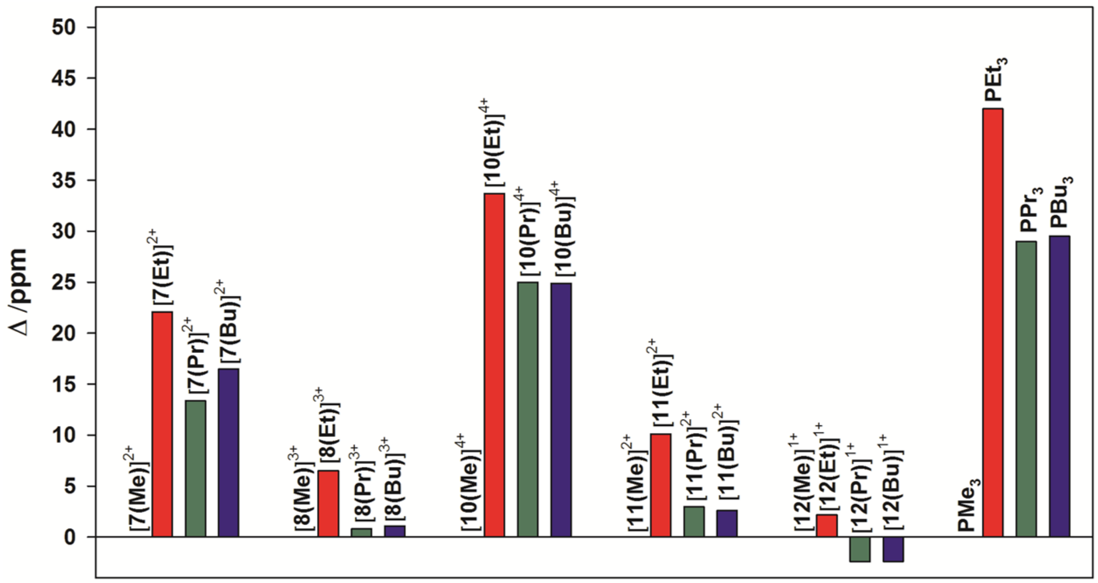

Figure S6. ^{31}P NMR chemical shifts of derivatives of [**7(R)**]$^{2+}$, [**8(R)**]$^{3+}$, [**10(R)**]$^{4+}$, [**11(R)**]$^{2+}$, [**12(R)**]$^{1+}$, and PR$_3$ (far right) relative to the PMe$_3$ containing derivative of each species (Δ, in ppm).

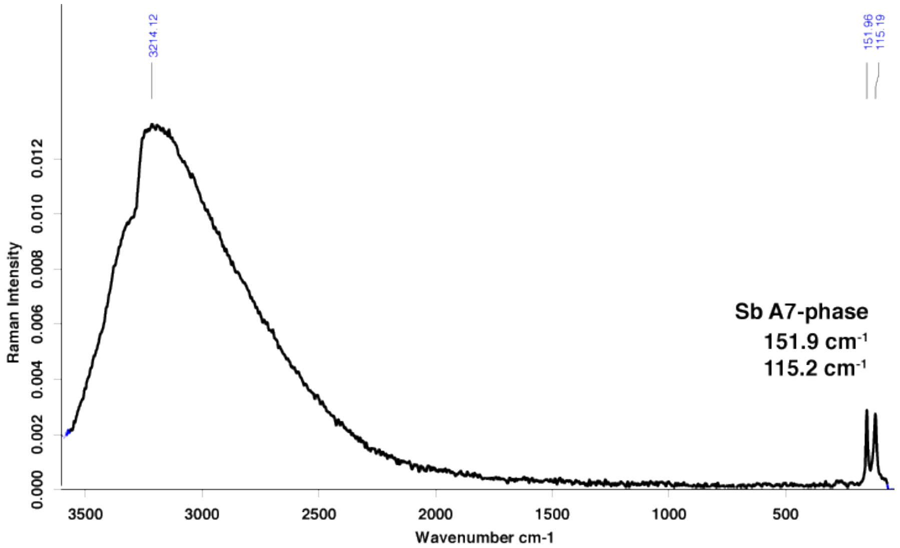

Figure S7. Raman spectrum (100 mW, 298 K) of black residue obtained upon thermolysis of [10(Me)][OTf]$_4$.

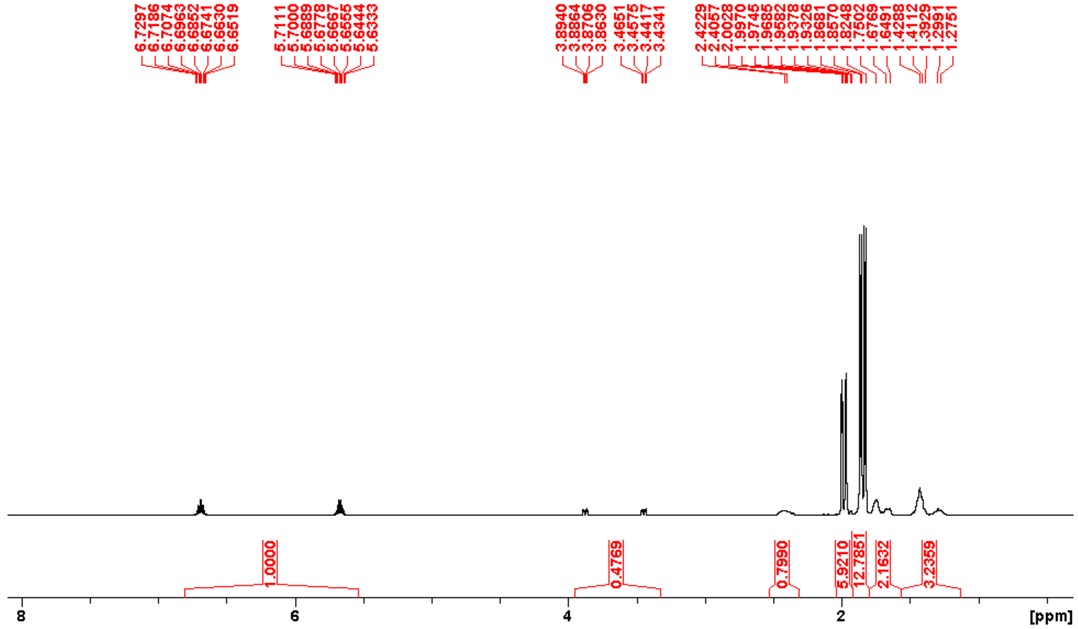

Figure S8. ^{1}H NMR spectrum (CD_3CN, 298 K, 500 MHz) of reaction mixture containing $CyPH_2$ and [**10(Me)**][OTf]$_4$ in a 2:1 ratio.

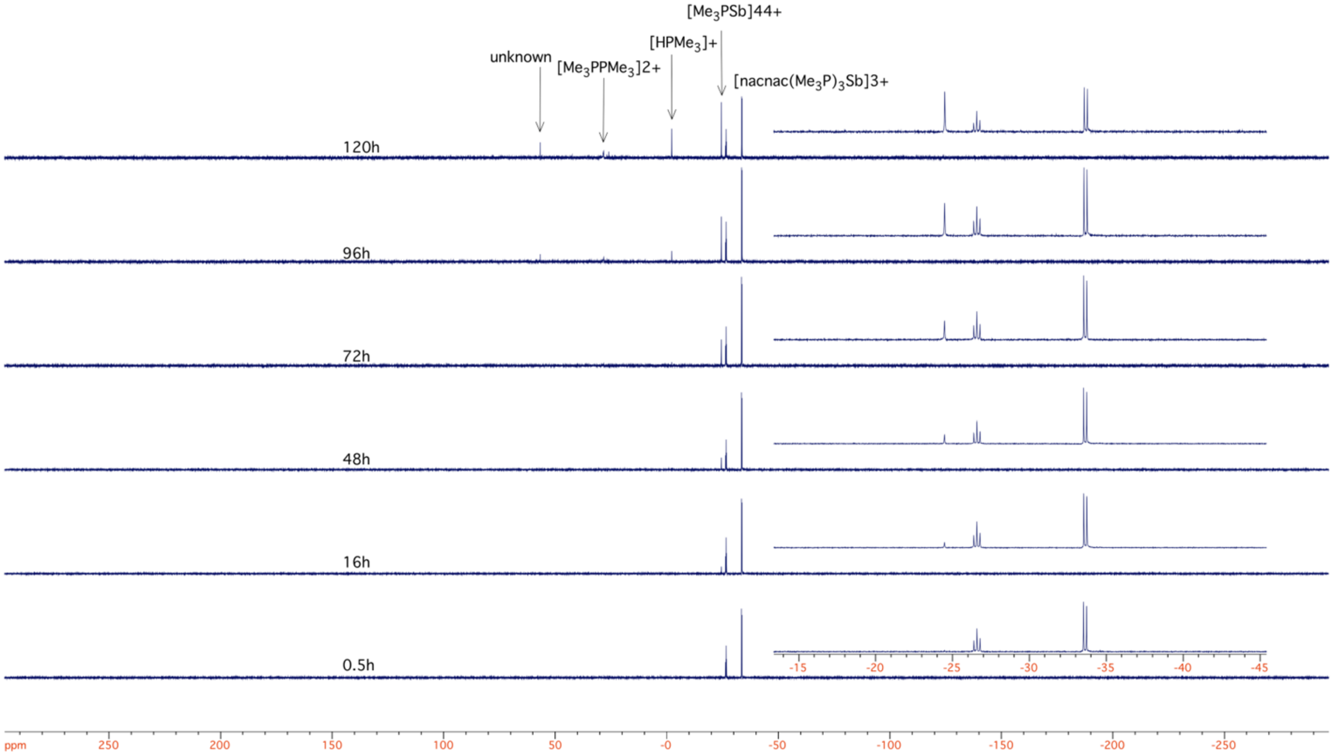

Figure S9. $^{31}P\{^{1}H\}$ NMR spectra (CH_3CN, 298 K) showing decomposition of a MeCN solution of [**15(Me)][**OTf]$_3$ at room temperature over 120 hours.

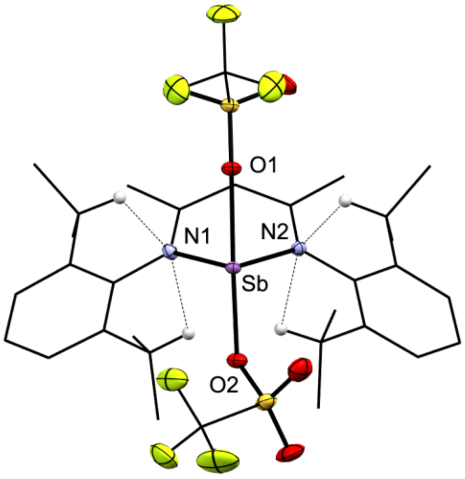

Figure S10. Molecular structure of (*nacnac*)Sb(OTf)$_2$ in the solid state. Non-essential hydrogen atoms have been omitted for clarity. Thermal ellipsoids are drawn at 30 % probability level. Bond lengths (Å) and angles (°) are as follows: N1-Sb = 2.071 (2), N2-Sb = 2.075 (2), O1-Sb = 2.287 (1), O2-Sb = 2.196(1), N1-Sb-N2 = 89.11(6), O1-Sb-O2 = 174.33(5), O1-Sb-N1 = 88.86(6), O1-Sb-N2 = 89.46(6), O2-Sb-N1 = 86.31(6), O2-Sb-N2 = 87.51(6).

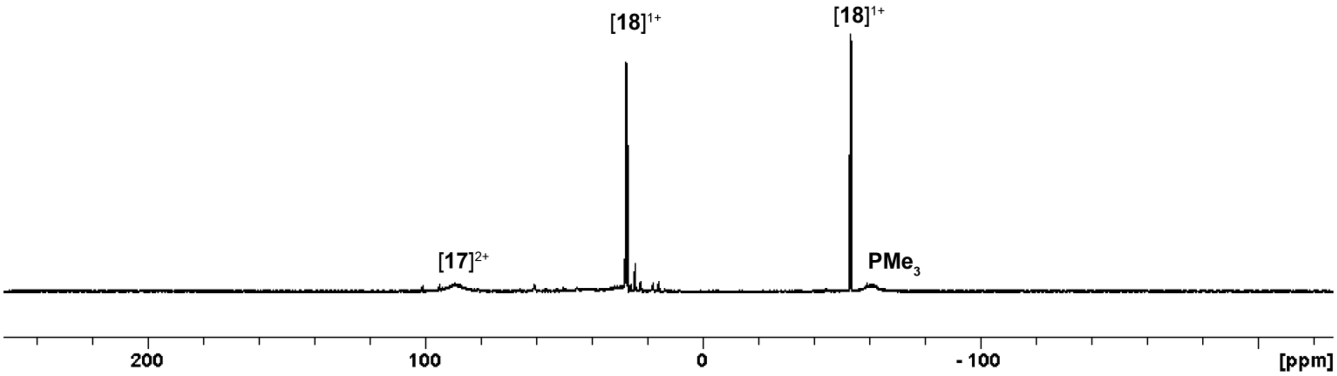

Figure S11. ^{31}P NMR spectrum (CH_3CN, 298 K, 202.5 MHz) of an equimolar reaction mixture containing *dmap* and [**11(Me)**]$^{2+}$ after 4 hours of stirring at room temperature.

Table S1. Crystallographic data for compounds [**13**][OTf]$_2$, [**11(Et)**][OTf]$_2$, [**12(Me)**][OTf], [**10(Et)**][OTf]$_4$•(MeCN), [**15(Me)**][OTf]$_3$•(MeCN), and [(*nacnac*)SbOTf$_2$].

	[**13**][OTf]$_2$	[**11(Et)**][OTf]$_2$	[**12(Me)**][OTf]	[**10(Et)**][OTf]$_4$•(MeCN)	[**15(Me)**][OTf]$_3$•(MeCN)	[(*nacnac*)SbOTf$_2$]
formula	$C_8H_{16}F_7O_6P_2S_2Sb$	$C_{14}H_{30}P_2S_2O_6F_6$	$C_4H_9O_3F_4PS$	$C_{58}H_{123}F_{24}NO_{24}P_8S_8Sb_8$	$C_{43}H_{71}F_9N_3O_9P_3S_3Sb_4$	$C_{31}H_{41}F_6N_2O_6S_2Sb$
M (g mol^{-1})	589.02	534.44	244.14	3152.81	1621.12	837.53
crystal system	monoclinic	monoclinic	monoclinic	Monoclinic	Triclinic	Monoclinic
space group	P2$_1$/n	P2$_1$/n	P2$_1$/m	P2$_1$/c	P-1	P2$_1$/c
a /Å	14.5029(6)	13.4271(9)	10.2147(7)	23.986(16)	11.4789(3)	12.3127(4)
b /Å	8.9034(4)	11.8532(8)	8.6511(6)	21.830(14)	11.9874(3)	17.4575(6)
c /Å	14.9927(6)	15.7504(11)	11.0323(7)	22.193(15)	26.0864(7)	17.0214(6)
α /°	90	90	90	90	91.8200(10)	90
β /°	97.2600(10)	110.9930(10)	94.5503(8)	106.570(14)	97.5640(10)	91.030(2)
γ /°	90	90	90	90	112.0740(10)	90
V /Å^3	1920.41(14)	2340.4(3)	971.83(11)	11138(13)	3284.57(15)	3658.1(2)
Z	4	4	4	4	2	4
T /K	173.15	173.15	173.15	173.15	173.15	173.15
crystal size /mm	0.21x0.15x0.13	0.36x0.12x0.08	0.43x0.30x0.21	0.21x0.17x0.16	0.31x0.19x0.16	0.31x0.26x 0.22
ρ /g cm^{-3}	2.037	1.517	1.669	1.880	1.639	1.521
F(000)	1152	1112	496	6168	1600	1704
radiation /Å	MoKα (λ = 0.71073)	MoKα (λ = 0.71073)	MoKα (λ = 0.71073)	MoKα (λ = 0.71073)	MoKα (λ = 0.71073)	MoKα (λ = 0.71073)
2*θ* range /°	3.682-54.608	3.432-52.762	3.704-56.594	1.78 -54.00	1.58 -54.00	2.33-60.00
μ /mm^{-1}	1.908	0.439	0.534	2.272	1.867	0.941
absorption correction	TWINABS	SADABS	SADABS	SADABS	SADABS	SADABS
reflections collected	4279	4792	2533	453078	64496	138828
reflections unique	3773	3709	2353	24284	14357	10665
R_{int}	0.0397	0.0271	0.0201	0.0551	0.0334	0.0537
residual density (e Å^{-3})	0.39<br>-0.33	0.315<br>-0.305	0.47<br>-0.28	3.390<br>-1.802	1.664<br>-1.163	2.033<br>-0.640
parameters	299	391	177	1205	825	443
G.O.F	1.023	1.030	1.055	1.225	1.173	1.046
R_1 [I > 2σ(I)]	0.0214	0.0372	0.0303	0.0560	0.0405	0.0271
wR_2 (all data)	0.0490	0.0961	0.0890	0.1485	0.1174	0.0684
CCDC No.	1040080	1040085	1040084	1040082	1040081	1040083

[1] Y. Ding, H. W. Roesky, M. Noltmeyer, and H. Schmidt, *Organometallics*, **2001**, *20*(6), 1190-1194.
[2] S. S. Chitnis, Y. Carpenter, N. Burford, R. McDonald, and M. J. Ferguson, *Angew. Chem. Int. Ed.*, **2013**, *52*, 4863-4866.
[3] Manuscript under review. A pdf version of the synthesis and characterization data is attached for reviewer's only.
